# Hyperspectral imaging benchmark based on machine learning for intraoperative brain tumour detection

**DOI:** 10.1038/s41698-023-00475-9

**Published:** 2023-11-14

**Authors:** Raquel Leon, Himar Fabelo, Samuel Ortega, Ines A. Cruz-Guerrero, Daniel Ulises Campos-Delgado, Adam Szolna, Juan F. Piñeiro, Carlos Espino, Aruma J. O’Shanahan, Maria Hernandez, David Carrera, Sara Bisshopp, Coralia Sosa, Francisco J. Balea-Fernandez, Jesus Morera, Bernardino Clavo, Gustavo M. Callico

**Affiliations:** 1 Research Institute for Applied Microelectronics, University of Las Palmas de Gran Canaria, Las Palmas de Gran Canaria, Spain; 2 Fundación Canaria Instituto de Investigación Sanitaria de Canarias (FIISC), Las Palmas de Gran Canaria, Spain; 3 Nofima, Norwegian Institute of Food Fisheries and Aquaculture Research, Tromsø, Norway; 4 Facultad de Ciencias, Universidad Autónoma de San Luis Potosí, San Luis Potosí, México; 5 Department of Biostatistics and Informatics, Colorado School of Public Health, University of Colorado Anschutz Medical Campus, Aurora, Colorado, USA; 6 Department of Pediatric Plastic and Reconstructive Surgery, Children’s Hospital Colorado, Aurora, Colorado, USA; 7 Instituto de Investigación en Comunicación Óptica, Universidad Autónoma de San Luis Potosí, San Luis Potosí, México; 8 Department of Neurosurgery, University Hospital Doctor Negrin of Gran Canaria, Las Palmas de Gran Canaria, Spain; 9 Department of Psychology, Sociology and Social Work, University of Las Palmas de Gran Canaria, Las Palmas de Gran Canaria, Spain; 10 Research Unit, University Hospital Doctor Negrin of Gran Canaria, Las Palmas de Gran Canaria, Spain

**Keywords:** CNS cancer, Surgical oncology, Brain imaging, Cancer imaging

## Abstract

Brain surgery is one of the most common and effective treatments for brain tumour. However, neurosurgeons face the challenge of determining the boundaries of the tumour to achieve maximum resection, while avoiding damage to normal tissue that may cause neurological sequelae to patients. Hyperspectral (HS) imaging (HSI) has shown remarkable results as a diagnostic tool for tumour detection in different medical applications. In this work, we demonstrate, with a robust k-fold cross-validation approach, that HSI combined with the proposed processing framework is a promising intraoperative tool for in-vivo identification and delineation of brain tumours, including both primary (high-grade and low-grade) and secondary tumours. Analysis of the in-vivo brain database, consisting of 61 HS images from 34 different patients, achieve a highest median macro F1-Score result of 70.2 ± 7.9% on the test set using both spectral and spatial information. Here, we provide a benchmark based on machine learning for further developments in the field of in-vivo brain tumour detection and delineation using hyperspectral imaging to be used as a real-time decision support tool during neurosurgical workflows.

## Introduction

In 2020, brain and central nervous system (CNS) cancer was the twelfth most common cancer in terms of mortality, with an estimated 308,102 incident cases, associated to 251,329 deaths worldwide for both sexes and all ages^1^. These numbers are expected to increase by 38.5% and 43.7% for incidences and mortality, respectively, for 2040^2^. In the young population under 35 years of age, it was the second most common cancer in terms of mortality (31,181 deaths) after leukaemia^1^, while in children under 14 years old, it was the second most common cancer in terms of both morbidity and mortality (24,388 incident cases/11,889 deaths) worldwide^1^. Particularly, brain tumours account for >90% of occurrence within CNS cancers, linked to high mortality and morbidity, especially in paediatric cases^3,4^.

Brain tumours are divided into primary and secondary (also called *metastatic*) tumours. Primary tumours appear in the brain, while secondary tumours appear elsewhere in the body and, then, metastasize to the brain^5^. Primary tumours are also divided into low-grade (LG) and high-grade (HG) according to their malignity. LG tumours include grades 1 and 2 (G1 and G2), while HG tumours correspond to grades 3 and 4 (G3 and G4), being glioblastoma (G4) the most frequent (~50%) and deadly (5-year survival rate of 5.5%) primary brain tumor^6^. The new grade Arabic numbering has been recently introduced in the 2021 WHO (World Health Organization) classification of CNS tumors^7^. Moreover, brain tumours can be intra-axial, which are located within the brain parenchyma and arise from the brain cells, or extra-axial, which are located outside the brain parenchyma and arise from the structures lining or surrounding it (e.g., meninges)^8^.

Surgical resection is the most common treatment for primary brain tumours, especially for diffuse gliomas, since the early and total resection of the tumour increase the overall survival rate (e.g., 5-year survival rate of 50% for diffuse astrocytoma and 81% for oligodendroglioma^6^). In this sense, the extent of resection increases the survival rates of patients with all types of gliomas. However, to achieve maximal resection, neurosurgeons need to determine the precise limits of the tumour during surgery using imaging-guiding techniques^9^. Additionally, neurosurgeons must avoid damaging normal tissue, which can lead to neurological deficits in patients and thus affect their quality of life (QoL)^10^.

Current intraoperative imaging guidance techniques have several limitations^9^. Image-guided stereotactic (IGS) neuronavigation is based on pre-operative imaging, such as standard magnetic resonance imaging (MRI), T1-weighted gadolinium-enhanced (T1G), T2 (T2w) or fluid attenuation inversion recovery (FLAIR). Nevertheless, IGS is affected by the brain shift phenomenon produced due to changes in tumour volume caused by craniotomy^11^. Intraoperative MRI (iMRI) requires specialised operating theatres and equipment, increasing the time and cost of surgery^12^. Ultrasound (US) is a less expensive alternative to iMRI that provides real-time imaging not affected by navigation inaccuracy or intraoperative changes (like in IGS). However, it has problems related to artefacts (blood, haemostatic material, bones, etc.) and requires long training periods to create high-quality images, resulting in 2D images that are difficult to interpret^13^. Intraoperative fluorescence imaging, such as 5-aminolevulimic acid (5-ALA) or fluorescein sodium (FS) are commonly used in brain surgeries for delineating tumour boundaries. Nonetheless, these fluorescence agents do not detect the majority of LG gliomas and must be orally administered to the patient, possibly producing side effects^14,15^. Consequently, there is still room for new research in imaging modalities and methods that could overcome these limitations, offering substitute or complementary approaches to the current state-of-the-art techniques^16^. There is a current necessity to develop new imaging acquisition and visualization systems to provide quick, detailed, accurate and highly personalised diagnostics for optimal decision-making during neurosurgical procedures, improving the outcomes in the QoL of the patient and reducing the errors, surgical times, and costs.

**Fig. 1:**
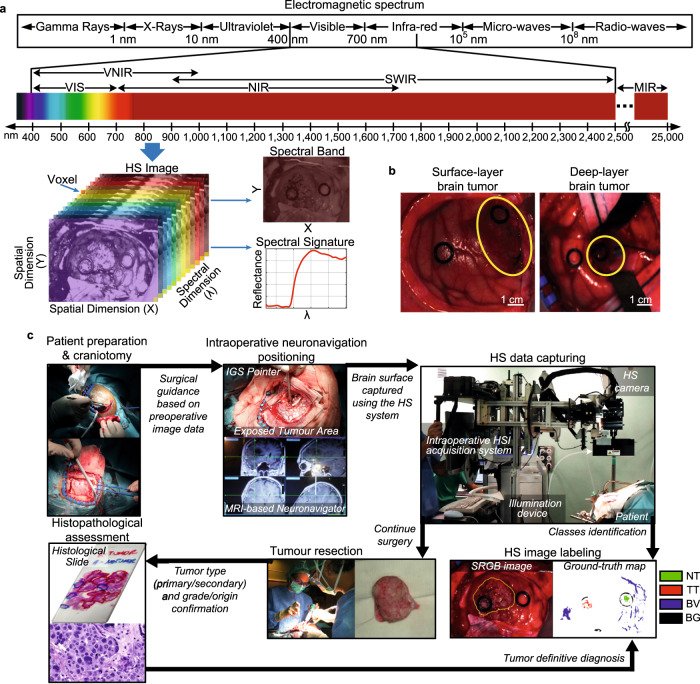
Proposed intraoperative HSI approach for surgical assistance. **a** HSI concept explanation. **b** Synthetic RGB images of a surface-layer tumour (left) and a deep-layer tumour (right). Tumour area is surrounded in yellow by a clinical expert. **c** HS data acquisition and labelling procedure during surgery. In the ground-truth map, red represents tumour tissue (TT) labelled pixels, green normal tissue (NT) pixels, blue blood vessel (BV) pixels, and black background (BG) pixels. Meanwhile, white represents non-labelled pixels. VIS Visual; VNIR Visual and Near Infrared; SWIR Short-Wave Infrared; MIR Mid-Infrared; SRGB Synthetic Red-Green-Blue.

In this sense, hyperspectral (HS) imaging (HSI) is an emerging technique capable of providing label-free, non-contact, near real-time, and minimally-invasive intraoperative guidance by using non-ionizing illumination and without employing any contrast agent^17^, hence being totally harmless for the patient. HS images are formed by hundreds of narrow spectral channels within and beyond the visual spectral range (Fig. 1a). This technique provides, for each pixel, a continuous spectrum that allows the identification of the tissue, material or substance present in the captured scene based on its chemical composition^18^.

In recent years, medical HSI has started to achieve promising results in many different specialities (e.g., oncology^19,20^, digital and computational pathology^21^, ophthalmology^22^, dermatology^23,24^ or gastroenterology^25,26^) through the utilization of cutting-edge artificial intelligence (AI) algorithms and thanks to the increased modern computational power^27,28^. Promising results are being achieved in different types of cancer using HSI^19^. Particularly, HSI has been widely studied in the literature for gastrointestinal cancer in both in-vivo and ex-vivo tissue samples, including stomach, liver, oesophagus, pancreas, and colorectal cancer^26^. For example, Tsai et al. presented a new method using HSI and deep learning (DL) to diagnose in-vivo oesophageal cancer, improving accuracy by 5% compared to RGB (red-green-blue) images^29^. In the field of head and neck cancer, Eggert et al. combined the use of HSI and DL to discriminate between healthy and tumour tissue in laryngeal, hypopharyngeal, and oropharyngeal mucosa, reaching an accuracy of 81%^30^. In addition, in the field of dermatology, this technology has been extensively used to study in-vivo skin cancer lesions^31,32^, however there is a lack of large, high-quality datasets of HS skin lesion images to develop trustworthy AI-based algorithms that will improve current results achieved with standard RGB images^23^. Additionally, HSI is becoming a tool not only for cancer detection, but also for the diagnosis of other diseases, such as biomarker discovery and validation^33^ or tissue perfusion measurement^34^. For example, it can provide an early diagnosis of diabetic retinopathy, as symptoms are not presented at the early stages of this disease^35^ or potentially identify biomarkers for the non-invasive detection of Alzheimer’s disease through in-vivo retinal HSI measurements^36^. Moreover, HSI has the potential to be used during awake brain surgery to identify eloquent brain areas adjacent to tumours (already explored in functional ultrasound imaging^37^).

Previous works from this group have evaluated, as a proof-of-concept, the use of HSI and data processing frameworks, particularly machine learning (ML) and DL algorithms, for intraoperative brain tumour detection and delineation using a limited set of images and patients, employing a leave-one-patient-out cross-validation, and focused in glioblastoma detection^38–40^. In this work, a more exhaustive spectral characterization of the different tissue and tumour types with an increased dataset is provided, as well as a more robust generation and validation of the classification results obtained using both the spectral and the spatial information for tumour delineation and identification targeting pathology-assisted surgery with real-time performance.

## Results

### Spectral characterization of brain tissue

**Fig. 2:**
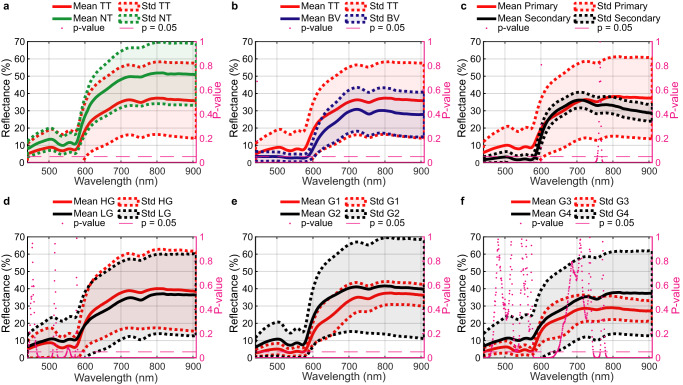
Spectral characterization of different brain tissue and tumour types. Mean (solid lines) and standard deviation (std) (dashed lines) of the entire labelled dataset after applying a basic pre-processing (calibration, extreme band noise removal, and noise filtering) and separated by classes, including the corresponding *p*-value (magenta dots) computed for each spectral channel using the paired two-sided Wilcoxon Rank Sum test at 5% of significance level between the two compared classes. **a** TT vs. NT class. **b** TT vs. BV class. **c** Primary vs. secondary tumours. **d** HG vs. LG primary tumours. **e** G1 vs. G2 primary tumours. **f** G3 vs. G4 primary tumours.

Sixty-two HS images obtained from 34 different patients (Supplementary Table 1) were captured and labelled to create ground-truth maps (GTs) following a specific protocol for data collection (Fig. 1b, c) (see Methods). Four classes were established: *tumour tissue* (TT), *normal tissue* (NT), *blood vessel* (BV), and *background* (BG). Raw HS data were pre-processed (see Methods) to standardize and reduce the noise of the spectral signatures. Statistical differences were found between all the medians of each spectral channel when comparing TT vs. NT (Fig. 2a) and TT vs. BV (Fig. 2b) using the paired two-sided Wilcoxon Rank Sum test at 5% of significance level. High standard deviation values were obtained in the spectral signatures due to the interpatient variability and also the different tumour types included in the database. Additionally, the intraoperative HS data acquisition during surgery is a complex procedure that can be affected, in some cases, by the non-flat brain surface (Fig. 1b). These irregular surfaces can affect the illumination conditions, and, hence, the image focus in certain areas, reducing the reflectance values and increasing the noise of the spectral signature respect to the more focused areas. For this reason, a complete pre-processing chain (see Methods) was applied to the HS data, where each spectral signature was normalized as minimum 0 and maximum 1 to only consider the shape of the signature in the processing algorithms computation. Additionally, a decimation process was applied to reduce the dimensionality of the data in the spectral dimension and, hence, the computational cost of the processing algorithms^41^.

**Fig. 3:**
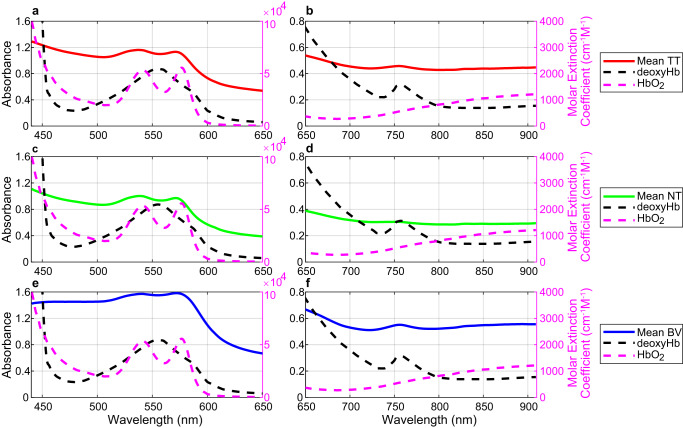
Spectral characterization of TT, NT, and BV classes and their relationship with HbO_2_ and deoxyHb. Mean absorbance values of the entire labelled dataset separated by classes (solid lines) after applying a basic pre-processing (calibration, extreme band noise removal, and noise filtering) and molar extinction spectra (dashed lines) of HbO_2_ and deoxyHb. **a** TT class between 440 and 650 nm. **b** TT class between 650 and 910 nm**. c** NT class between 440 and 650 nm **d** NT class between 650 and 910 nm. **e** BV class between 440 and 650 nm. **f** BV class between 650 and 910 nm.

The mean spectral signatures of TT, NT, BV were converted to absorbance values (Fig. 3) to be represented and compared with the molar extinction coefficient of oxyhaemoglobin (HbO_2_) and deoxyhaemoglobin (deoxyHb)^42^. Absorbance values of all classes increase between 500 and 600 nm (Fig. 3a,c,e), due to the existence of two HbO_2_ absorbance peaks ( ~ 540 and ~575 nm) and one deoxyHb absorbance peak (~555 nm) in this spectral range^43^. Particularly, HbO_2_ peaks in BV are not detected (Fig. 3e), probably because we labelled veins and arteries indistinctly, involving HbO_2_ and deoxyHb characteristics. Higher absorbance values were found in TT with respect to NT, but lower than BV. Moreover, an absorbance peak was found at ~760 nm (Fig. 3b, f) related to deoxyHb^44,45^. Our spectral data reveal that the contribution of deoxyHb is the highest in BV (Fig. 3f), having a lower contribution in TT (Fig. 3b). However, this contribution is not found in NT (Fig. 3d). This difference between NT and TT could be mainly related to the lack of oxygenation in the brain tissue affected by cancer^46^.

### Spectral characterization of different brain tumour types

As stated in the introduction section, brain tumours can be subdivided into different subtypes depending on their origin (primary/secondary) or the grade of malignity in the case of primary tumours. Regardless of tumour grade and origin, there is an absorbance peak (reflectance valley) around 760 nm (Fig. 2c–f) related to deoxyHb^44^. Secondary tumours present lower standard deviation values than primary ones (Fig. 2c). However, this fact can be related to the reduced number of patients affected by secondary tumours in our database, and the reduced number of labelled pixels with respect to the primary type. Despite of this, statistical differences between the medians of each spectral channel were found at 440–599, 602–756 and 769–909 nm spectral ranges. HG and LG primary tumours present similar reflectance and standard deviation values (Fig. 2d). Nonetheless, statistical differences were found at 466–510, 522–549, 559–572 and 580–909 nm spectral ranges (Fig. 2d). Considering the tumour grades of primary tumours (Supplementary Fig. 1), statistical differences were found between the medians of all spectral channels of G1 and G2 tumours (Fig. 2e), whereas in the case of G3 and G4 tumours (Fig. 2f), only the 440–460, 578–644, 745–764 and 779–909 nm spectral ranges were found to be statistically different.

### Brain tissue classification based on spectral information

The HS data collected intraoperatively was used as input for ML and DL-based algorithms (i.e., random forest (RF), k-nearest neighbours (KNN) using Euclidean (KNN-E) and Cosine (KNN-C) distances, support vector machines (SVMs) using the linear (SVM-L) and radial basis function (SVM-RBF) kernels, and a two-layer deep neural network (DNN)) and unmixing-based methods (i.e., linear extended blind end-member and abundance extraction (EBEAE) and nonlinear extended blind end-member and abundance extraction (NEBEAE)) to generate classification models capable of distinguishing between the four different classes (TT, NT, BV, and BG). Labelled data were used to train and optimize the hyperparameters of the algorithms and to quantitatively evaluate the results on the test set (see Methods). Due to the high computational cost required to train the ML/DL algorithms, training data were reduced to 1000, 2000, and 4000 pixels per class, following the method proposed in a previous work^41^. In general, during the optimization process using the macro F1-Score metric independently to the training data reduction used (Supplementary Figs. 2–4), the results tend to stabilize at a certain hyperparameter for all the five folds (Supplementary Table 2–4).

**Fig. 4:**
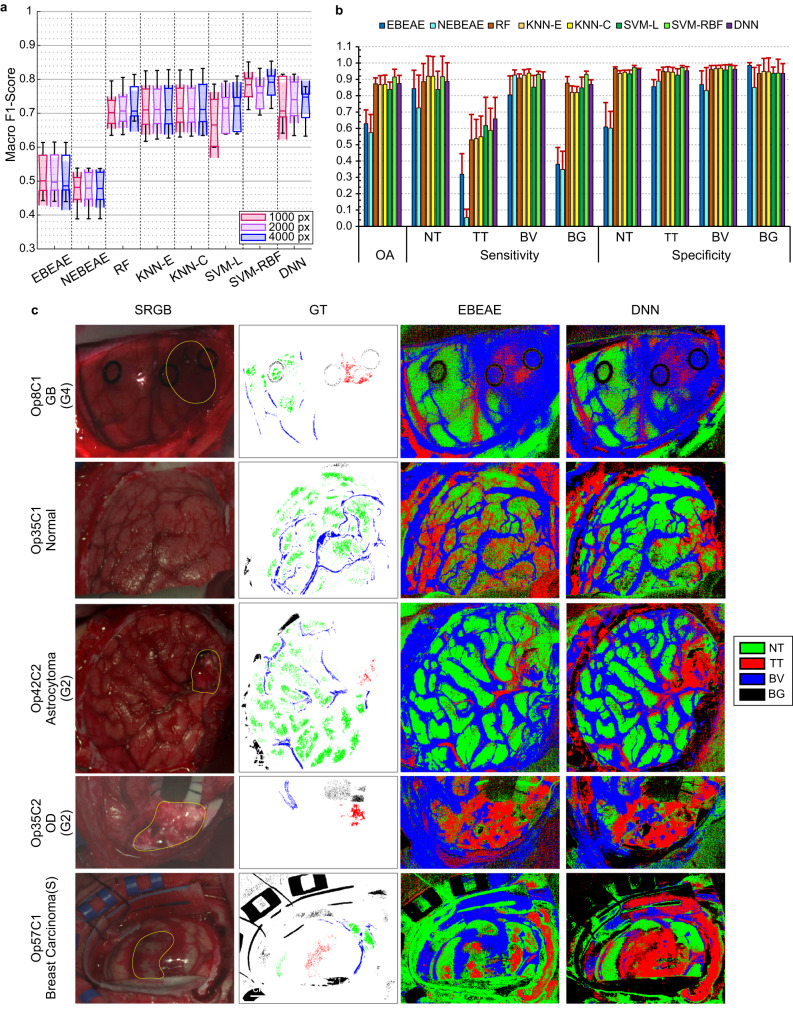
Spectral classification results of brain tissue. **a** Boxplots of the macro F1-Score results of the validation set for each training data reduction and each classifier, including the 5 folds using the optimal hyperparameters in each classifier. In the plot, the centre line, the box limits and the whiskers represent the median, the upper and lower quartiles and the 1.5× interquartile range, respectively. Two medians are significantly different at the 5% significance level if their intervals (shaded colour areas) do not overlap. **b** Average OA, sensitivity, and specificity results of the validation set from the 5 folds using the data reduction of 1000 pixels per class (error bars represent the standard deviation). **c** Examples of SRGB images, GT maps and supervised classification maps generated using the EBEAE and DNN algorithms with the optimal hyperparameters from different tumour types of the validation set. Approximate tumour areas were surrounded in yellow lines on the SRGB image by the operating surgeon according to the intraoperative neuronavigation and the definitive pathological diagnosis of the resected tissue. Rubber ring markers were employed in some cases (e.g., Op8C1) to indicate the area where the biopsies for pathology were resected. Opx Operation number x; Cy Capture number y.

No statistically significant differences were found between the three training data reductions (Fig. 4a). Hence, the use of 1,000 pixels per class allowed to reduce the time required for training the model (particularly for the SVM-based implementations) without compromising the classification performance. For this reason, we selected this training data reduction for the subsequent experiments. Additionally, our results show that statistically significant differences were found between the unmixing-based methods and the ML-based ones, obtaining lower classification performance. The highest median macro F1-Score result was obtained with the SVM-RBF model (78.4 ± 5.1%), but no statistically significant differences were observed between this algorithm and the others (except for EBEAE and NEBEAE). The highest average overall accuracy (OA) was also reached by the SVM-RBF (91.5 ± 4.7%), but the highest TT sensitivity (65.9 ± 13.1%) was obtained with the DNN (Fig. 4b). Average specificity results were >90% for the ML and DL-based approaches.

Qualitative results, extracted from the validation set and obtained after applying the supervised classification model (generated using 1,000 pixels per class and the optimal hyperparameters) to the entire HS image, show the pixel-wise identification of both labelled and non-labelled pixels (Fig. 4c and Supplementary Fig. 5). As expected, according to the quantitative results (Fig. 4a,b), the unmixing-based methods (EBEAE and NEBEAE) increase the number of false positives and false negatives in the classification maps, particularly in Op35C1 employing EBEAE, where the normal tissue is identified as tumour, and Op57C1 using NEBEAE, where tumour areas are identified as normal tissue. The remaining classifiers achieve more consistent results, although the SVM-based and DNN algorithms improve the identification of the tumour areas in Op42C2 and Op57C1 (only using SVM-L and DNN).

### Brain tumour identification and delineation based on spatial-spectral information

**Fig. 5:**
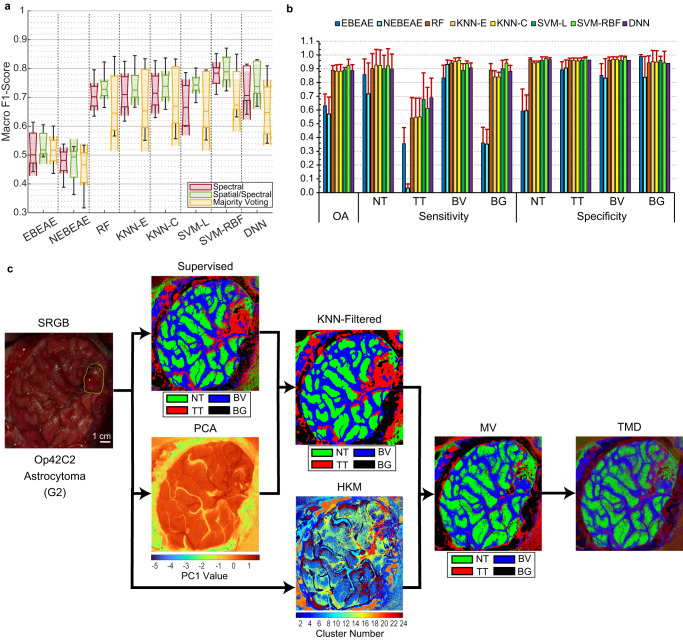
Quantitative and qualitative results at the different stages of the proposed framework in the validation set. **a** Boxplots of the macro F1-Score of the validation set using the eight different classifiers at the three different stages. In the plot, the centre line, the box limits and the whiskers represent the median, the upper and lower quartiles and the 1.5× interquartile range, respectively. Two medians are significantly different at the 5% significance level if their intervals (shaded colour areas) do not overlap. **b** Average OA, sensitivity, and specificity results of the validation set from the 5 folds using the *Spatial/Spectral* approach (error bars represent the standard deviation). **c** Example of the output maps at the different stages of the proposed framework from Op42C2 of the validation set (based on the DNN as supervised algorithm using the optimal hyperparameters).

Following the approach proposed in a previous work^38^, the use of the spatial information available in the HS images was included to evaluate the possible improvement of the classification results and to reduce the false positives found in the supervised classification maps computed based only on the spectral information (see Methods). We have compared the quantitative results of the validation set (Fig. 5a) using only the spectral information (*Spectral*), applying the KNN filtering to include also the spatial information (*Spatial/Spectral*) and combining the spatial-spectral supervised classification with an unsupervised segmentation through a majority voting (MV) approach (*Majority Voting*). Our results reveal that the inclusion of the spatial information increases the median macro F1-Score results (0.4-7.7%), reducing the standard deviation (0.2–3.7%), in all algorithms, except for the unmixing-based approaches. However, no statistical differences were found between these results. Additionally, it is worth noticing that the *Majority Voting* results achieved lower median results and increased the std. Nonetheless, this lower performance could be motivated by the construction of the output classification map in the MV approach, which is obtained by considering only the majority class assigned to each cluster of the unsupervised hierarchical k-means (HKM) map. At the *Spatial/Spectral* stage, the SVM-RBF reached the highest average OA (92.3 ± 4.6%), but the DNN obtained the best average TT sensitivity (68.9 ± 14.3%), closely followed by the SVM-L algorithm (67.7 ± 19.3%) (Fig. 5b).

The qualitative results of each step of the proposed algorithm have been analysed, where the supervised map represents, as an example, the classification map generated using only spectral information with the DNN method (Fig. 5c and Supplementary Fig. 6). The PCA (Principal Component Analysis) map represents, in a false colour intensity map, the first principal component where the more important information contained in the HS image is relocated in a low dimensional space. For example, in Op8C1 (Supplementary Fig. 6), the tumour area is partially highlighted with more intensity values (between the two rubber ring markers on the right of the image). The KNN-Filtered map offers a smoothed version of the supervised map, where the spatial properties of the HS image are used (by combining the information of the probability maps generated by the supervised classifier and the PCA map). This approach reduces the granularity of the supervised map, providing more homogeneous class regions. This *Spatial/Spectral* classification was combined with an unsupervised segmentation (HKM map) that identifies 24 different regions (or clusters) in the HS image according to their similar spectral characteristics, providing a very accurate delineation of different structures but without any identification of the tissue, material or substance that each cluster represents. For this reason, the information provided by the HKM map was merged with the KNN-Filtered map by means of a MV approach^38^, where each cluster is labelled by the majority class within it. In the MV map (Fig. 5c), the boundaries between different class regions are determined by the HKM map, while the identification of each cluster class is defined by the KNN-Filtered map. However, in these maps, only the information relative to the class with the majority number of pixels in each cluster is shown. Hence, as a surgical visualization tool, we proposed to combine the information provided by the three maximum probability values (classes NT, TT, and BV) of the MV approach, by mixing the RGB colours in each cluster according to the percentage of pixels covered by each class in such cluster (i.e., the R channel corresponds to the percentage of TT pixels, the G channel to NT pixels, and the B channel to BV pixels). For example, a cluster represented by a bright red, green or blue colour denotes it belongs to only one single class (TT, NT, or BV, respectively). In contrast, any other colour represents a combination of classes in a cluster (e.g., purple colour represents a mixture between TT and BV classes that commonly happens in certain blood vessels, hypervascularized areas or extravasated blood, see Op42C2, Op35C2 or Op57C1 in Fig. 5c and Supplementary Fig. 6). This resulting map is called three maximum density (TMD) map^38^ (Fig. 5c).

**Fig. 6:**
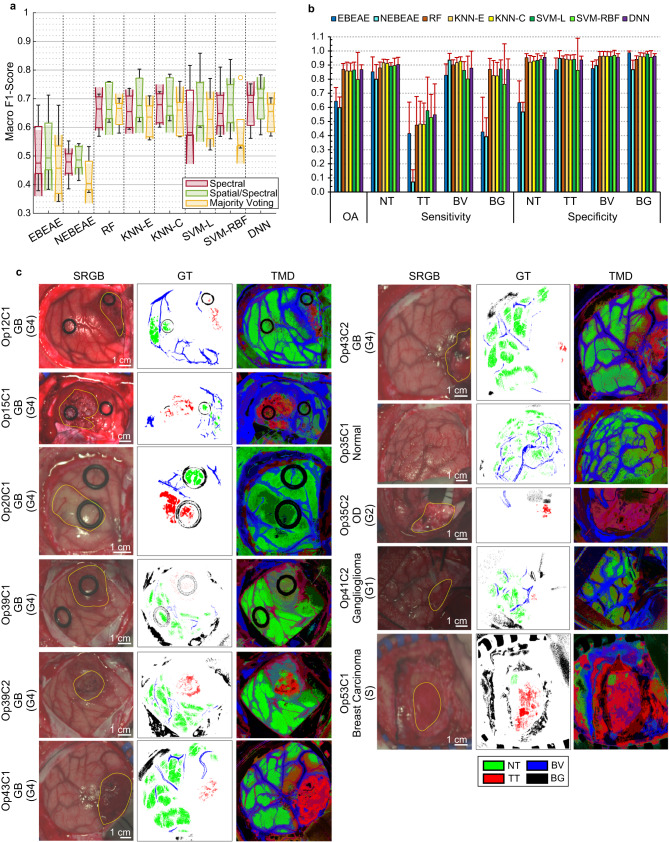
Quantitative results at the different stages of the proposed framework and qualitative TMD classification maps in the test set. **a** Boxplots of the macro F1-Score of the test set using the eight different classifiers at the three different stages. In the plot, the centre line, the box limits and the whiskers represent the median, the upper and lower quartiles and the 1.5× interquartile range, respectively. Two medians are significantly different at the 5% significance level if their intervals (shaded colour areas) do not overlap. **b** Average OA, sensitivity, and specificity results of the test set from the 5 folds using the *Spatial/Spectral* approach (error bars represent the standard deviation). **c** Examples of SRGB images, GT maps and TMD maps from different tumour types (based on the DNN as supervised algorithm using the optimal hyperparameters).

After performing all the analysis and hyperparameter optimizations of the algorithms using the validation set, the test sets of the different k-folds were evaluated (Fig. 6a). Quantitative results of the macro F1-Score metric show, as expected, a performance reduction in the test set of 0.5-1% respect to the validation one, providing the best median score of 70.2 ± 6.3% using the DNN algorithm in the *Spatial/Spectral* approach. Similar average OA results are obtained using SVM-L (86.6 ± 5.5%) and DNN (86.8 ± 3.4%) as supervised classifiers, while a slight increase of the SVM-L average TT sensitivity (57.8 ± 23.7%) respect to the DNN (54.7 ± 21.9%) is obtained (Fig. 6b). Specificity average results are in general >90% in all ML and DL-based approaches for all classes.

Some examples of the TMD maps of the test set (Fig. 6c) show that the glioblastoma cases (Op12C1, Op15C1, Op39C2, Op43C1, and Op43C2) delineate in red the tumour areas, as expected by neurosurgeons (marked in yellow over the synthetic RGB images). Particularly, Op15C1 presents some decoloured red/orange/purple areas that could represent the infiltrative nature of glioblastoma tumours in the surrounding tissue. Moreover, the surrounding blue areas could be related to the hypervascularized tissue that surrounds the tumour, also including the blood vessels in such regions (Op15C1 in Fig. 6c). The same fact can be visualized in Op12C1, Op43C1, and Op43C2. In the case of Op20C1 and Op39C1, the tumour is somehow revealed but not as a red area, since the tumours are located in a deep layer of the brain tissue. Op20C1 has not an additional image captured after the resection started, since the tumour resection in such location of the brain could cause serious damages and side effects to the patient, and, additionally, the tumour boundaries were not clear enough to perform a secure and effective resection. For such reason, the operating surgeon decided not to operate the patient, prevailing the QoL of the patient. On the contrary, after Op39C1 was captured, the operating surgeon continued the tumour resection, and a second image (Op39C2) was captured during resection, where it is possible to observe the correct delineation of the tumour area in a bright red colour. This was also the case of Op43, but before starting the resection, the tumour was clearly visualized in the brain surface, showing a possible infiltration in the surrounding tissue (orange/purple colour in the upper-left part of the tumour area).

Moreover, we qualitatively evaluated some examples of test cases not related to high-grade gliomas (Fig. 6c). Firstly, Op35C1 presents a healthy brain surface, since the tumour was in a deep layer, where no false positives are present in the parenchymal area, only those related to extravasated blood surrounding the parenchymal area. In Op35C2 and Op41C2, it is possible to observe that the proposed algorithm can identify not only high-grade tumours but also low-grade tumours, a G2 oligodendroglioma and a G1 ganglioglioma, respectively. Finally, secondary tumours are also detected by the proposed algorithm, as shown in Op35C1 where a metastatic breast carcinoma is identified, although some false positives surrounding the parenchymal area are also presented. These false positives could be produced because of the low quality of the image, where an optimal focus was not achieved.

### Comparison with previous related works in HSI and other techniques

Different previously published works used the first data campaign from the in-vivo HS brain database employed in this work. In 2018, the hybrid framework used in this study, which combined supervised and unsupervised machine learning methods to perform a classification based on spatial and spectral information was presented^38^. However, the study only employed 5 HS images from 5 patients achieving an overall accuracy of 99.7% using an intra-patient methodology, which commonly provides unrealistic optimistic results. Later, the same framework was tested using the complete first data campaign (36 HS images from 22 patients), but only a qualitative analysis of the results was performed^39^. In 2019, a DL approach was proposed to identify glioblastoma tumours obtaining an OA of 80% following an inter-patient approach using 26 HS images from 16 patients, where only 8 HS images contained tumour tissue pixels labelled, evaluating only these HS images in the test set using a leave-one-patient-out cross-validation methodology^40^. In 2020, another research employed a blind linear unmixing method (EBEAE) to identify glioblastoma as a low computational time cost alternative. This work employed 26 HS images from 16 patients, where only 6 HS images contained tumour tissue pixels labelled, achieving an OA of 76.1% using a leave-one-patient-out cross-validation methodology^47^. Later, a nonlinear unmixing approach based on a multilinear mixture model (NEBEAE) was employed, performing an intra-patient validation process with the same database. Using 2 HS images from different patients the method achieved an OA of 97.9%^48^, providing again unrealistic results that can be employed in a real-world scenario. A method based on the fusion of multiple deep models was proposed by Hao et al. to use the spectral and spatial information to identify glioblastoma. The proposed method achieved an OA of 96.6% for four-class classification and OA of 96.3% for glioblastoma identification adopting a leave-one-patient-out cross validation technique using 7 HS images from 5 patients^49^.

Urbanos et al. presented a HS acquisition system to acquire and process HS images during surgical environment using a snapshot HS camera able to capture 25 bands along the spectral range from 655 to 975 nm^50^. An HS database composed of 13 HS images from 12 patient was employed to train SVM, RF, and convolutional neural network (CNN) classifiers achieving the best OA result using SVM (60.0%) and an intra-patient approach. Using the same HS database, Martín-Pérez et al. performed a comparison between non-optimized models with optimized models and employing an intra-patient approach with 10 HS images from 9 patients^51^. This comparison was performed using SVM and RF algorithms and three different optimization methods: *grid search*, *random search*, and *Bayesian optimization*. The study showed that the RF results did not provide significant improvement when the model was optimized with any of the three optimization methods. However, the optimized SVM model improved the tumour identification. Sancho et al. presented SLIMBRAIN^52^, a modification of the HS system presented by Urbanos et al. that incorporated augmented reality using a LiDAR (Light Detection and Ranging or Laser imaging Detection and Ranging) camera. The classification results obtained from the HS images were overlapped with the RGB point cloud captured by the LiDAR camera and presented in an augmented reality visualization.

In the field of surgical microscopes combined with HS cameras, Mühle et al. integrated an HS camera into a neurosurgical microscope for brain tumour resections^53^. In this proof of concept, a single HS image was analysed using an RF classifier to discriminate between healthy tissue, malignant tissue, vessels, and background. Using a 5-fold stratified cross-validation methodology, the RF classifier achieved an overall accuracy of 99.1%, providing again unrealistic results to be extrapolated in real-world scenarios. Puustinen et al. developed an operating microscope-integrated HSI system for microneurosurgery as a monitoring tool during neurosurgical operations^54^. As a proof of concept, two HS images of in-vivo glioma tumours were labelled and used to train and test different ML algorithms, achieving the best OA of 98.3% and an accuracy to identify the glioma class of 97.7% using the light gradient boosting machine algorithm. Giannantonio et al. presented an intraoperative HS system based on a surgical microscope coupled to a visual and near-infrared (VNIR) camera^55^. In this study, the authors presented a dataset of low-grade gliomas (grade 1 and 2) composed of 18 HS images from 5 patients. Different algorithms were used (RF, SVM, and MLP), obtaining an OA of 92.0% using an intra-patient methodology.

Table 1 summarize and compare the current studies found in the literature which employs HSI for in-vivo brain tumour detection. In particular, a significant number of studies are based on small datasets and focus primarily on the identification of high-grade tumours. Moreover, these studies are developed to utilize an intra-patient framework. In contrast, our work uses an extensive and diverse database that includes the four tumour grades and different tumour types, both primary and secondary. The use of this database and the validation framework based on an inter-patient approach and a three-way data partition (*training*, *validation*, and *test*) combined with a 5-fold cross-validation approach (see Methods section), allow us to obtain robust results mitigating the risk of overfitting commonly caused by AI-based algorithms.

**Table 1:** Summary of the studies found in the literature which employs HSI for in-vivo brain tumour detection.

Reference	Year	HSI System Type	Wavelengths (nm)	#Bands	#Patients	#HS Images	Tumour Grade	Patient Variability	Validation Methodology	OA (%)	Tumour Accuracy (%)
Fabelo et al.^38^	2018	Pushbroom	400–1000	826	5	5	4	Intra-patient	10-fold CV	99.7	99.5
Fabelo et al.^68^	2018	Pushbroom	400–1000	826	26	43	1, 2, 3, and 4	Inter-patient	Training/test (85%/15%)	n/a	n/a
Fabelo et al.^40^	2019	Pushbroom	400–1000	826	16	26	4	Inter-patient	LOPO-CV	80.0	42.0
Cruz-Guerrero et al.^69^	2020	Pushbroom	400–1000	826	16	26	4	Inter-patient	LOPO-CV	76.1	n/a
Hao et al.^53^	2021	Pushbroom	400–1000	826	5	7	4	Inter-patient	LOPO-CV	96.6	96.3
Mühle et al.^70^	2021	Pushbroom - Surgical Microscope	500–1000	100	1	1	3	Intra-patient	Training/test (80%/20%) + 5-fold CV	99.1	n/a
Urbanos et al.^54^	2021	Snapshot	655–975	25	12	13	3 and 4	Intra-patient	Training/test (80%/20%) + 5-fold CV	60.1	73.0
Campos-Delgado et al.^52^	2022	Pushbroom	400–1000	826	2	2	4	Intra-patient	n/a	97.7	n/a
Martin-Perez et al.^55^	2022	Snapshot	655–975	25	9	10	3 and 4	Intra-patient	Training/test (80%/20%)	n/a (AUC = 98.6%)	n/a
Sancho et al.^56^	2023	Snapshot	655–975	25	12	13	3 and 4	Intra-patient	Training/test (80%/20%)	n/a (AUC = 95.2%)	n/a (AUC = 95.1%)
Puustinen et al.^58^	2023	Snapshot - Surgical Microscope	500–900	n/a	1	2	1 and 3	Intra-patient	Training/test (75%/25%)	98.3	97.7
Giannantonio et al.^71^	2023	Snapscan - Surgical Microscope	470–780	104	5	18	1 and 2	Intra-patient	Training/test (70%/30%)	92.0	n/a
This work	2023	Pushbroom	400–1000	826	34	61	1, 2, 3, and 4	Inter-patient	3-way data partition (60%/20%/20%) + 5-fold CV	86.8 ± 3.4	57.8 ± 23.7

*OA* Overall accuracy, *LOPO* Leave-one-patient-out, *CV* Cross-validation, *AUC* area under the curve, *n/a* Not Available

As stated in the introduction, intraoperative fluorescence imaging is able to provide real-time tumour identification in surgical practice making use of a contrast agent previously administered to the patient. In neurosurgical procedures, three fluorescent contrast agents have been studied: FS, indocyanine green (ICG), and 5-ALA^56^. The protoporphyrin IX (PpIX) fluorescence provides intraoperative visual differentiation between healthy and malignant tissue during resection of high-grade gliomas^57^. On the one hand, Molina et al. evaluated the use of 5-ALA-induced PpIX fluorescence in malignant glioma surgery^58^. A total of thirty-two patients with grades 3 and 4 brain tumours were included in the study, performing 128 fluorescence margin biopsies. The sensitivity of fluorescence to detect tumour tissue was 70.8%. On the other hand, Valdes et al. evaluated the use of PpIX in low-grade gliomas using seventy-three biopsies from 12 patients with grades 1 and 2 brain tumours^59^. In this study, the authors identified significant concentrations of PpIX which imply the potential for detecting low-grade tumours. Using ICG, Cho et al. evaluated thirty-six patients with grade 4 brain tumours, performing 78 biopsies^60^. The study achieved a sensitivity of 97.0% for the presence of NIR fluorescence to detect tumour tissue. Additionally, Lee et al. evaluated the use of ICG fluorescence agent during surgery using a NIR camera^61^. The study analysed 14 patients with grade 1 and 2 meningiomas. The NIR fluorescence system achieved a sensitivity of 96.4% to identify tumour tissue after analysing 46 biopsies performed to such patients. With FS, Acerbi e*t al*. evaluated thirteen patients with high-grade glioma^62^. The study analysed 50 biopsies achieving a sensitivity result of FS in identifying tumour tissue was 80.8%. Sweeney et al. performed a study of 98 patients with grade 4 tumours who underwent surgical resection using FS^63^. A sensitivity of 62.0% was achieved in the identification of tumour tissue. These studies are summarized in Supplementary Table 5.

Currently it is not possible to make a fair comparison between the results obtained from HSI systems and intraoperative fluorescence imaging systems, due to the lack of rigorous clinical studies to evaluate the actual accuracy of HSI systems. However, it could be very useful to carry out this comparison in future clinical studies by using HSI systems in real environments during brain surgical procedures to test their usability, safety, efficacy, and efficiency respect to the tools currently employed.

### Preliminary post-hoc interpretability of ML models

**Fig. 7:**
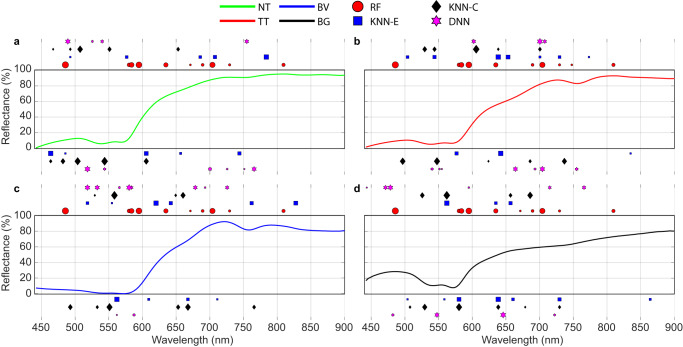
Post-hoc interpretability using LIME approach. Graphical representation of the ten most relevant features identified for the classification models by the RF, KNN-E, KNN-C and DNN algorithms using the training set of the first fold in each class. **a** Average spectral signature of the NT class. **b** Average spectral signature of the TT class. **c** Average spectral signature of the BV class. **d** Average spectral signature of the BG class. In the plot, the size of the markers represents the level of importance computed by LIME (higher size is related to higher importance). Positive coefficients are represented at the top of the chart while negative coefficients are shown at the bottom. RF is only evaluated with positive predictor importance values.

In the healthcare domain, ML models cannot be considered as “black box” models and need to be explainable and interpretable in order to provide a rationale behind the decisions made by the classifiers to identify a certain instance^64^. This can be carried out by using explainable artificial intelligence, such as LIME (local interpretable model-agnostic explanations)^65^, SHAP (Shapley additive explanations)^66^ or TreeExplainer^67^, among others. As a preliminary study, we have employed the LIME as a model-agnostic post-hoc approach to highlight the most relevant wavelengths considered by 4 out of the 6 ML models employed in this study for each class. In order to simplify this preliminary analysis, as an example, we have examined the ML models of RF, KNN-E, KNN-C, and DNN trained with the training set of the fold 1 established in this study. The LIME implementation was executed in the MATLAB Statistics and Machine Learning Toolbox version 12.2 (R2021b), using the ML model as the “black box” input. The number of important predictors to be identified was set to the maximum number of features of the HS data (i.e., 128 spectral bands). The synthetic dataset was composed by 5000 samples. Figure 7 shows the 10 most relevant spectral bands identified by LIME extracted from the 128 spectral bands for each model and class. Positive coefficients are represented at the top of the chart and negative coefficients at the bottom, where the different symbols represent the different ML models, and the higher size is related to higher importance. The average spectral signature of each class from the training set is also represented in the chart. The results show that the initial (<464 nm) and last (>835 nm) spectral bands are, in general, not included in the 10 most important spectral bands for all ML models. Nonetheless, the remaining wavelengths are considered relevant, especially those associated with the HbO_2_ (~540 and ~575 nm) and deoxyHb ( ~ 555 nm and ~760 nm) absorbance peaks. It is worth noticing that each ML model identifies different important spectral bands, mainly due to the different strategies employed by each classifier to extract the features from the HS data during the training process. However, in some cases, the same spectral bands (or adjacent bands) are highlighted by all classifiers. Supplementary Fig. 7 shows the coefficient values and the related specific wavelengths of these 10 most relevant bands for each classifier and class.

## Discussion

This work demonstrates the high potential of HSI for in-vivo identification of brain tumour tissue and its boundaries during neurosurgical operations. We have developed an intraoperative customized HS acquisition system, employed in three data acquisition campaigns, to collect 61 HS images of exposed brain surface from 34 different patients that underwent surgery due to brain cancer or another disease that required surgery. Using this extended database with respect to previous works^38–40,47,48^, we have analysed the spectral characteristics of the brain tissue (normal and tumour) and blood vessels, and the different tumour types according to their malignancy grades (G1 to G4) and origin (primary and secondary), performing a statistical analysis between all the medians of each spectral channel when comparing the different classes and tumour grades and origins. Moreover, a robust 5-fold cross-validation approach was used to evaluate eight different processing algorithms, first using only spectral information, and then using both spatial and spectral information following a processing framework that we previously developed^38^.

The spectral-based classification results obtained using the validation set (Fig. 4a) showed that SVM-based and DNN methods provided the best macro F1-Score results, although no statistical differences were found among the other classifiers (except for the unmixing-based methods, which provided less accurate results). The qualitative results (Fig. 4c and Supplementary Fig. 5) demonstrate the ability of the proposed HSI-based system to identify not only high-grade gliomas (Op8C1), but also other low-grade tumours (Op42C2 and Op35C2) and secondary tumours (Op57C1). Moreover, these results show the capability of HSI to accurately highlight the vascularization of the brain surface, being especially remarkable in Op35C1 and Op42C2.

It is worth noticing that HS images captured in suboptimal acquisition conditions, such as a lack of correct focus or illumination, can introduce inappropriate spectral signatures for training the algorithms and can produce inaccurate classification maps. This limitation is particularly evident in deep-layer tumours (Supplementary Fig. 8a), where it was not possible to correctly focus the entire area of interest by using our pushbroom-based HSI system. In the case of Op37C2 (Supplementary Fig. 8a), due to uncertainty at the time of labelling the tumour pixels in the centre of the image, only NT, BV, and BG classes were labelled. The average spectral signatures (Supplementary Fig. 8b) reveal an acquisition problem, possibly related to a lack of proper illumination, as the reflectance values in the three classes decrease dramatically in the infrared range (>700 nm). However, the DNN method seems to overcome this handicap and correctly identify the tumour area even using this non-optimal HS image.

The inclusion of spatial information improved the macro F1-Score medians respect to using only spectral information, although no statistical differences were found between these results (Fig. 5a). After performing the hyperparameter optimization process using the validation set, the test data of each k-fold were processed providing both quantitative and qualitative results (Fig. 6). The processing framework based on the DNN algorithm in the *Spatial/Spectral* approach provided a macro F1-Score of 70.2 ± 7.9%, representing, as expected, a performance reduction of 3.6% respect to the validation results. Qualitative test results demonstrate the ability of the proposed framework to identify not only HG gliomas (e.g., glioblastoma), but also LG and secondary tumours (e.g., G2 oligodengroglioma, G1 ganglioglioma, metastatic breast carcinoma) (Fig. 6c) and also extra-axial tumours (e.g., G1 meningioma).

The processing of the test dataset allowed us to identify some HS image cases where the data acquisition conditions were not optimal, producing some errors in the classification results (Supplementary Fig. 9a), which may degrade the quantitative results of the test sets. We found that in Op55C1 and Op55C2 the classification results identified most of the pixels as tumour, and only some parts related to background (Supplementary Fig. 9a). After evaluating the spectral signatures of the labelled pixels in such HS images, we found some differences in the infrared region (from 700 to 900 nm) with respect to the other images. This unusual behaviour was found also in Op56C2, where there is a decrease in the reflectance values of the labelled spectral signatures in the same infrared region (Supplementary Fig. 9b), also producing wrong classification results where the parenchymal area is identified as background (Supplementary Fig. 9a). The low sensitivity of the HS sensor in this spectral range, coupled with a possible misalignment of the light beam with the lens (due to an improper focusing), could lead to this decrease in reflectance.

Despite these limitations, we have demonstrated with a robust classification validation approach, the potential benefits of HSI for brain tumour tissue identification, targeting a diagnostic support system for guiding neurosurgical interventions in real-time. In previous works, we demonstrated that it is possible to achieve near real-time HS data processing using graphical processing units, achieving processing times of ~6 s^68^. The proposed intraoperative HSI-based acquisition system must be optimized in further works by reducing the HS camera size, employing a snapshot-based HSI technology (which is able to capture the entire HS cube in a single shoot, providing also real-time performance) and integrating it into a surgical microscope. This new experimental setup will guarantee an improvement of the HS image quality to solve the focus problems, especially for deep-layer tumours. Additionally, an extensive clinical validation of the proposed framework must be carried out, employing a large number of patients and a multi-centre trial. This clinical validation will perform a comprehensive pathological analysis of the entire tumour area outlined by the TMD map (especially in the boundaries between tumour and the surrounding normal tissue), as well as correlate the results with the MRI information to verify that the system can adequately identify tumour infiltration into normal brain tissue, especially in HG gliomas. Additionally, the relation between the improvement of the patient outcomes and the use of the proposed system during the surgery could be studied through the clinical validation.

## Methods

### Study population

**Fig. 8:**
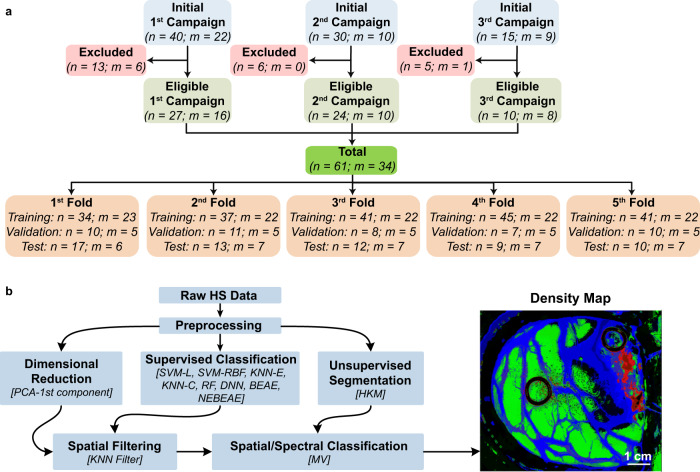
Data partition and proposed processing framework. **a** Patient/image flow scheme of this work and data partition. n Number of HS images, m number of patients. **b** Proposed processing framework to generate the density maps for intraoperative pathology-assisted surgery.

All patients over 18 years of age, with primary or secondary brain tumours, undergoing brain surgery at the University Hospital of Gran Canaria Doctor Negrín (Spain) who were capable of giving informed consent for this study protocol before the surgery. Patients were enrolled in three different data acquisition campaigns (Fig. 8a) carried out between March 2015 to June 2016 (First Campaign), October 2016 to April 2017 (Second Campaign) and July 2019 to October 2019 (Third Campaign). Written informed consent was obtained from all the participant subjects, including the publication of any potentially identifiable images or data. Consent for publication was obtained from all individuals who appears in the figures. The study protocol and consent procedures were approved by the Research Ethics Committee of the University Hospital Doctor Negrin (Ref 130069 for the first and second campaigns, and Ref 2019-001-1 for the third campaign). All the research methodologies were performed in accordance with relevant guidelines/regulations.

### Study procedure

HS images were captured following the procedure (Fig. 1c) previously described in detail^69^. First, a craniotomy was performed to the patient by using IGS neuronavigation and then, the durotomy was accomplished to expose the brain surface. Next, the acquisition system was placed over the patient’s brain to acquire the HS image. In some particular cases, rubber ring markers were placed over tumour and normal tissue areas according to the IGS system information to further identify the tissue type. After that, tumour tissue was resected for neuropathological evaluation to achieve the definitive diagnosis of the tumour. When possible, more than one HS image were acquired while the tumour was being resected.

### In-vivo hyperspectral brain database

HS images were manually cropped to select the region of interest where the parenchymal area was exposed. Afterwards, the data were labelled by using the information provided by the neuropathologists and the knowledge of the operating surgeons, using a semi-automatic labelling tool based on the spectral angle mapper (SAM) algorithm developed for this purpose^69^. The GT maps (Fig. 1c) were composed by four classes: TT (red colour), NT (green colour), BV (blue colour), and BG (black colour). White pixels in the GT maps represent the non-labelled pixels, as only those with a high confidence of belonging to a particular class were labelled. Several images in the database do not contain tumour pixels due to the impossibility of performing a reliable labelling or due to the patient underwent surgery for another pathology, such as a blood clot or epilepsy.

Sixty-one HS images of exposed brain surface from 34 different patients were included in the experiment after excluding several HS images due to the inadequate capturing conditions (Fig. 8a). Supplementary Table 1 summarizes the number of patients (identified as Op$x$: operation number) and images (identified as C$y$: capture number) included from each data campaign, as well as their image dimensions, number of labelled pixels and the definitive pathological diagnosis.

### Clinical data collection

A total of 61 HS images were acquired from 34 adult patients with brain tumours. The summary of the patient demographics and clinic data is shown in Table 2, while the detailed information is presented in Supplementary Table 6. Ages ranged from 30 to 73 years, with a median age of 51.5 years. Among these patients, there were 21 males and 13 females. Of these 34 patients, 28 (82.4%) had a primary tumour. The most frequency primary grade was the G4 (44.1%, $n=15$), followed by G1 and G2 (14.7%, $n=5$ each one), while the 8.8% ($n=3$) of the tumours were G3. The remaining 6 (17.6%) tumours were secondary: 3 from breast carcinoma, 2 from lung (one adenocarcinoma and one carcinoma), and 1 from kidney (renal carcinoma). Most of tumours were located in the right temporal lobe (23.5%, $n=8$), followed by the left frontal and right parietal lobes (20.6%, $n=7$ each).

**Table 2:** Summary of patient demographic and tumour characteristics.

Variable [patients with no missing values/Total patients]	Characteristic	Total (n)	%
Sex [34/34]	Male	21	61.8
	Female	13	38.2
Age [33/34]	Median	51.5	-
	Range	30–73	-
Tumour Type [34/34]	Primary	28	82.4
	Secondary	6	17.6
Primary Tumour Grade [28/34]	WHO Grade 1	5	14.7
	WHO Grade 2	5	14.7
	WHO Grade 3	3	8.8
	WHO Grade 4	15	44.1
Metastasis [6/34]	Breast	3	8.8
	Lung	2	5.9
	Kidney	1	2.9
Location [34/34]	Right Frontal Lobe	3	8.8
	Left Frontal Lobe	7	20.6
	Right Parietal Lobe	7	20.6
	Left Parietal Lobe	4	11.8
	Right Temporal Lobe	8	23.5
	Left Temporal Lobe	1	2.9
	Right Occipital Lobe	2	5.9
	Left Occipital Lobe	1	2.9
	Cerebellum	1	2.9

### Intraoperative hyperspectral acquisition system

HS images of the in-vivo brain were obtained intraoperatively using a custom HS acquisition system (Fig. 1b) previously described^39^ and later improved^70^. In brief, the system was composed by a VNIR HS pushbroom camera (Hyperspec^®^ VNIR A-Series, Headwall Photonics Inc., Fitchburg, MA, USA) able to capture 826 spectral channels in the 400–1000 nm spectral range, having a spectral resolution of 2–3 nm and a maximum spatial resolution of 741 × 1004 pixels, due to the pushbroom mechanism for the data acquisition process^39^. An illumination system based on a quartz tungsten halogen (QTH) lamp of 150 W coupled to a fibre optic cold light illuminator was employed, avoiding the incidence of the high temperatures of the QTH lamp in the exposed brain tissue. The working distance between the HS camera lens and the brain surface was 40 cm, with a pixel size of 128.7 µm and a maximum acquisition time of 60 s.

### HS data pre-processing

Raw HS images were pre-processed to avoid the influence of ambient illumination and dark currents of the HS sensor, and to reduce dimensionality and noise following the procedure previously described^41^. In brief, a raw HS image was calibrated following Eq. (1), where ${CI}$ is the calibrated image, ${RI}$ is the raw image, and ${WI}$ and ${DI}$ are the white and dark reference images, respectively. The white reference was acquired in the same illumination conditions that the raw HS image was captured, using a standard white reference tile that reflects the 99% of the incident light. The dark reference was obtained by keeping the camera shutter closed, being used to correct the dark currents produced by the HS sensor. Finally, a data smoothing approach based on a moving average filter was applied for reducing the high-frequency noise. Each smoothed value was averaged using a window of five data points. Moreover, the extreme spectral channels of the HS cube (the first 56 and the last 126 spectral channels) were removed due to the low capabilities of the HS sensor in these channels, which produces high noise in the spectral signatures^69^. At this point, the HS cube is formed by 645 channels with an operating bandwidth between 440.5–909.1 nm. HS cubes with this pre-processing chain were used for the spectral characterization of the different tissue and tumour types. The spectral signatures were also converted to absorbance ($A$) following Eq. (2) to compare the spectra of the different classes with the molar extinction coefficients of deoxyHb and HbO_2_, where $R$ is the reflectance value and $\lambda$ represents each wavelength.

In addition to this pre-processing, a dimensionality reduction of the HS cube was performed by decimating the spectral channels to reduce redundant information in the HS data due to the high dimensionality, also allowing a drastic reduction of the execution time of the processing algorithms without losing diagnostic performance. As studied in a previous work^41^, the optimal sampling interval was 3.61 nm, allowing the number of spectral channels to be reduced to 128. Finally, the spectral signatures were normalized independently to minimum and maximum values of [0, 1].

1 $${CI}=\frac{{RI}-{DI}}{{WI}-{DI}}$$

2 $$A(\lambda )=-\log (R(\lambda ))$$

### Supervised classification algorithms

ML algorithms used in this work were based on SVM, RF, and KNN classifiers, while the DL algorithm employed was a two-layer one-dimensional DNN. Moreover, two unmixing-based algorithms were studied (EBEAE and NEBEAE) using their MATLAB implementations^48,71^. SVMs are widely used for classification and regression purposes^72^. The objective of this classifier is to separate different data classes by finding out the best separation hyperplane with a maximum margin. In this study, the optimal hyperplane was computed employing linear and RBF kernels. The LIBSVM library was used for the SVM-L and SVM-RBF implementations^73^. The hyperparameter to be optimized for the SVM-L was the cost (*C*) parameter, which controls the decision limit that separates the positive and negative classes, while for SVM-RBF the hyperparameters optimized were cost and gamma ($\gamma$). RF is based on decision trees, identifying the new data class by taking a vote of their predictions from an aggregation of decision trees^74^. The optimization of the RF model was performed by searching for the most appropriate number of trees (*T*). KNN compares each incoming sample with all their neighbours using a distance metric to find the closest neighbors^75^. For the KNN classifier, we employed the Euclidean and Cosine distance metrics and the hyperparameter to be optimized in each case was the number of nearest neighbours (*N*). The MATLAB (R2021b) Statistics and Machine Learning Toolbox version 12.2 was employed for the RF and KNN-E and KNN-C implementations. The DNN was composed by two hidden layers, followed by a batch normalization layer, using the rectified linear unit as an activation function. The learning rate was established as 0.1, and the network was trained for 300 epochs. The output size (*L*) of the hidden layers was optimized. The MATLAB (R2021b) Deep Learning Toolbox version 14.3 was used for the DNN implementation. This DNN structure was studied in a previous work and compared with a two-dimensional CNN implementation, achieving the DNN the best performance^40^. The EBEAE is employed in non-negative datasets using a linear mixing model to perform the estimation of characteristic spectral endmembers and their abundances^71^. The NEBEAE is a nonlinear version of EBEAE, capable of quantifying non-linear optical interactions during the acquisition process, which is also robust against noise^48^. In both cases, different hyperparameters can be modified to adjust the similarity among endmembers (ρ) and the entropy of the abundances ($\gamma$). These algorithms have been previously used to identify glioblastoma tumour in pathological slides and in-vivo tissue using HS data^40,47,48,71,76^. In this case, the characteristic endmembers were estimated by the EBEAE and NEBEAE algorithms, respectively. The estimation process was performed using the labelled pixels from the training set. The representative number of endmembers was two for NT, two for TT, one for BV and three for BG, while the ρ hyperparameter was set as 0.3 for NT, 0.2 for TT, and 0.01 for BG^47^. The endmember of the BV class was obtained by calculating the average of all labelled pixels in that class. In both algorithm the entropy weight ($\gamma$) hyperparameter was optimized during the estimation of the complete abundance matrix.

### Three-way data partition and k-fold cross-validation

To correctly evaluate the classification performance of the proposed approach, a three-way data partition was carried out at patient level, dividing the HS database into training (60%), validation (20%), and test (20%) sets. Additionally, five different folds were created to achieve more robust results due to the limited number of patients. This data partition was performed randomly using the patients’ identifiers as instances, where each patient could have more than one HS image (Fig. 8a and Supplementary Table 7). Labelled data were employed to train the classification models (*training set*), to optimize their hyperparameters (*validation set*), and to quantitatively evaluate the results using unseen HS data (*test set*). The hyperparameter optimization of each algorithm was performed in each fold independently, evaluating the results with their respective validation sets and using the macro F1-Score metric and performing a coarse search (Supplementary Fig. 2–4). The optimal hyperparameters were selected using the best macro F1-Score result of each fold without considering the BG class.

### Training data reduction approach

Due to the high computational cost required to train several of the employed classifiers, a methodology based on K-Means algorithm^41^ was used to reduce the number of pixels in the training set (see Supplementary Table 8), balancing the classes, avoiding the inclusion of redundant information, and drastically reducing the training execution time. In this approach, K-means clustering is applied independently to each class of labelled pixels in the training set to obtain 100 different clusters ($K=100$) per class empirically selected (in this work, 400 clusters in total related to the four classes: NT, TT, BV, and BG). Thus, 100 centroids corresponding to a particular class are obtained. To reduce the original training data set, these centroids are used to identify the most representative pixels of each class using the SAM algorithm. For each centroid, only the $n$ most similar pixels are included in the reduced training set. In this work, three different number of similar pixels were evaluated ($n\in \{10,20,40\}$), generating three different training sets composed by 1000, 2000, and 4000 pixels per class (100 centroids × $n$ pixels). The total number of labelled pixels in the HS images from the validation and test sets was used for the quantitative evaluation of the processing framework (see Supplementary Table 8). This approach was evaluated in a previous work, where different metrics were compared with respect to the completed training set. The results obtained in such work revealed that the OA did not present a relevant change between using the completed and reduced training sets, however, the accuracy of TT class improved up to ~20% and the execution time when training the classification model was drastically decreased (an speedup of ~48 × ) when using the reduced set^41^.

### Proposed processing framework for TMD map generation

The spatial-spectral approach is based on a combination of a dimensionality reduction, a supervised classifier, a spatial filtering, an unsupervised segmentation, and a MV algorithm to merge the results from both supervised and unsupervised approaches (Fig. 8b). This approach was employed in previous works^38,40^ to prove that the use of the spatial information available in the HS images helps to improve the classification results and to reduce the misclassified pixels found in the supervised classification maps created using only the spectral information. In this work, the PCA algorithm was employed for dimensionality reduction^39^, obtaining a one-band representation of pre-processed HS image (Fig. 5c). The spatial filtering aims to improve the supervised classification including the spatial features. The KNN filtering algorithm was employed using the previously studied parameters ($\lambda =1$ and $K=40$)^38^ and a window size of 8 rows using the Euclidean distance^77^. The probability maps from the supervised classifier and the one-band representation are the inputs of this algorithm. The HKM algorithm^38^ was used as the unsupervised segmentation method to identify *K* different clusters into the HS images ($K=24$ according to a previous work^38^). Finally, the MV algorithm is used to merge the results obtained from the spatial-spectral supervised classification and the unsupervised segmentation, using a colour gradient approach to create the TMD maps^38^. MATLAB (R2021b) Statistics and Machine Learning Toolbox version 12.2 was employed to implement the PCA and KNN filtering algorithms.

### Performance metrics

The classification performance was evaluated using macro F1-Score (Eq. 3), OA (Eq. 4), sensitivity (Eq. 5), and specificity (Eq. 6) metrics, where ${TP}$ are true positives, ${TN}$ are true negatives, ${FN}$ are false negatives, and ${FP}$ are false positives. Macro F1-Score was computed with the mean of F1-Score per class (Eq. 7), where $i$ is the class index and $N$ the number of classes. BG class was not considered to obtain the macro F1-Score result. Additionally, spectral characterization results were statistically analysed using a paired two-sided Wilcoxon Rank Sum test at the 5% significance level.

3 $${Macro}\,F1-{Score}=\frac{1}{N}\mathop{\sum }\limits_{i=0}^{N}F1-{Scor}{e}_{i}$$

4 $${Specificity}=\frac{{TN}}{{TN}+{FP}}$$

5 $${OA}=\frac{{TP}+{TN}}{{TP}+{TN}+{FP}+{FN}}$$

6 $${Sensitivity}=\frac{{TP}}{{TP}+{FN}}$$

7 $$F1-{Score}=\frac{2{\rm{\cdot }}{TP}}{2{\rm{\cdot }}{TP}+{FP}+{FN}}$$

### Reporting summary

Further information on research design is available in the Nature Research Reporting Summary linked to this article.

### Supplementary information

Supplementary Information

REPORTING SUMMARY

## Data Availability

The authors declare that all data supporting the results of this study are available within the paper and its Supplementary Information. The datasets generated during the current study are available, under reasonable request, through https://hsibraindatabase.iuma.ulpgc.es/.

## Code availability

Custom MATLAB (version R2021b) codes are available on GitHub: https://github.com/HIRIS-Lab/InvivoBrainHSI-Benchmark.
