# Supplementary material for: Hyperspectral imaging benchmark based on machine learning for intraoperative brain tumour detection
Source: NPJ Precis Oncol. 2023 Nov 14;7:119. doi: 10.1038/s41698-023-00475-9 (PMC10646050; doi:10.1038/s41698-023-00475-9)
Supplement: Supplementary file 1 — Supplementary Information [file 41698_2023_475_MOESM1_ESM.pdf]

Supplementary Information for:

Hyperspectral Imaging Benchmark based on Machine Learning for Intraoperative Brain Tumour Detection

Raquel Leon[1*,+], Himar Fabelo[2,1*,+], Samuel Ortega[3,1], Ines A. Cruz-Guerrero[4], Daniel Ulises Campos-Delgado[4,5], Adam Szolna[6], Juan F. Piñeiro[5], Carlos Espino[6], Aruma J. O'Shanahan[6], Maria Hernandez[6], David Carrera[6], Sara Bisshopp[6], Coralia Sosa[6], Francisco J. Balea-Fernandez[7,1], Jesus Morera[6], Bernardino Clavo[8,2], and Gustavo M. Callico[1]

[1]Research Institute for Applied Microelectronics, University of Las Palmas de Gran Canaria, Las Palmas de Gran Canaria, Spain.
[2]Fundación Instituto de Investigación Sanitaria de Canarias (FIISC), Las Palmas de Gran Canaria, Spain.
[3]Nofima, Norwegian Institute of Food Fisheries and Aquaculture Research, Tromsø, Norway.
[4]Facultad de Ciencias, Universidad Autónoma de San Luis Potosí, San Luis Potosí, México.
[5]Instituto de Investigación en Comunicación Óptica, Universidad Autónoma de San Luis Potosí, México.
[6]Department of Neurosurgery, University Hospital Doctor Negrin of Gran Canaria, Las Palmas de Gran Canaria, Spain.
[7]Department of Psychology, Sociology and Social Work, University of Las Palmas de Gran Canaria, Las Palmas de Gran Canaria, Spain.
[8]Research Unit, University Hospital Doctor Negrin of Gran Canaria, Las Palmas de Gran Canaria, Spain.
[*]Corresponding authors: slmartin@iuma.ulpgc.es; hfabelo@iuma.ulpgc.es; [+]these authors contributed equally to this work.

List of Supplementary Figures

Supplementary Figure 1. Average spectral signature of the entire labelled dataset for each primary tumour grade. *G1: Grade 1; G2: Grade 2; G3: Grade 3; G4: Grade 4.* 4

Supplementary Figure 2. Coarse search representation of the macro F1-Score results using the data reduction of 1,000 pixels per class for the hyperparameter optimization of each classification algorithm using the validation set. Red dots and asterisks represent the optimal value. *N: Number of trees; K: Number of nearest neighbours; γ: Lambda; C: Cost.* 5

Supplementary Figure 3. Coarse search representation of the macro F1-Score results using the data reduction of 2,000 pixels per class for the hyperparameter optimization of each classification algorithm using the validation set. Red dots and asterisks represent the optimal value. *N: Number of trees; K: Number of nearest neighbours; γ: Lambda; C: Cost.* 6

Supplementary Figure 4. Coarse search representation of the macro F1-Score results using the data reduction of 4,000 pixels per class for the hyperparameter optimization of each classification algorithm using the validation set. Red dots and asterisks represent the optimal value. *N: Number of trees; K: Number of nearest neighbours; γ: Lambda; C: Cost.* 7

Supplementary Figure 5. Examples of synthetic RGB (SRGB) images, ground truth (GT) maps and supervised classification maps generated using the eight algorithms with the optimal hyperparameters from different tumour types of the validation set. Approximate tumour areas were surrounded in yellow line on the SRGB image by the operating surgeon according to the intraoperative neuronavigation and the definitive pathological diagnosis of the resected tissue. Rubber ring markers were employed in some cases (e.g., Op8C1) to indicate the area where the biopsies for pathology were resected. In the GT maps and classification maps, red represents tumour class, green normal class, blue blood vessel class, and black background class. *Opx: Operation number x; Cy: Capture number y*..8

Supplementary Figure 6. Example of SRGB images and output maps from different tumour types of the validation set at the different stages of the proposed framework (based on the DNN as supervised algorithm using the optimal hyperparameters). Approximate tumour areas were surrounded in yellow line on the SRGB image by the operating surgeon according to the intraoperative neuronavigation and the definitive pathological diagnosis of the resected tissue. In the GT, supervised, KNN-filtered, and MV maps, red represents tumour class, green normal class, blue blood vessel class, and black background class. PCA maps represent the value of the first principal component (blue color represents the minimum value and red the maximum). HKM maps represent 24 different clusters and colors have no meaning. *Opx: Operation number x; Cy: Capture number y*. 9

Supplementary Figure 7. Ten most relevant features identified using the LIME approach for the classification models by the RF, KNN-E, KNN-C and DNN algorithms using the training set of the first fold in each class. 10

Supplementary Figure 8. Examples of the limitations related to deep-layer tumours. a, Example of synthetic RGB images, GT maps and supervised classification maps created using the EBEAE and DNN algorithms with the optimal hyperparameters from a deep-layer tumour captured in non-optimal conditions in the validation set. Approximate tumour areas were surrounded in yellow on the SRGB image by the operating surgeon according to the intraoperative neuronavigation and the definitive pathological diagnosis of the resected tissue. b, Average spectral signatures (solid lines) of the GT pixels from a. In the ground-truth map and classification maps, red represents tumour class, green normal class, blue blood vessel class, and black background class. *Opx: Operation number x; Cy: Capture number y*. 11

Supplementary Figure 9. Examples of the limitations of the proposed framework after processing the test set. a, Example of SRGB images, GT maps and TMD maps (based on the DNN algorithm) from HS images captured in non-optimal conditions in the test set. Approximate tumour areas were surrounded in yellow on the SRGB image by the operating surgeon according to the intraoperative neuronavigation and the definitive pathological diagnosis of the resected tissue b, Average spectral signatures (solid lines) of the GT pixels from a. *Opx: Operation number x; Cy: Capture number y*. 12

List of Supplementary Tables

Supplementary Table 1. Summary of the in-vivo HS human brain image database, including number of pixels labelled of each class, spatial and spectral dimension, and the diagnosis. *NT: Normal; TT: Tumour; BV: Blood vessel; BG: Background; S: Secondary; P: Primary; G1: Grade 1; G2: Grade 2; G3: Grade 3; G4: Grade 4; DL: Deep layer tumour. Opx: Operation number x; Cy: Capture number y.* 13

Supplementary Table 2. Coarse search to optimize the hyperparameters of the processing algorithms to classify the data based on the spectral information using the validation and training set with 1000 pixels per class. *HP: Hyperparameter; I: Initial value; S: Step value; F: Final value; γ: Lambda; C: Cost; T: Number of trees; K: Number of nearest neighbours; L: Size of the output layers; EBEAE: Extended blind end-member and abundance extraction; NEBEAE: Nonlinear extended blind end-member and abundance extraction; RF: Random forest; KNN-E: K-nearest neighbour with Euclidean distance; KNN-C: K-nearest neighbour with cosine distance; SVM-L: Support vector machine with linear kernel; SVM-RBF: Support vector machine with radial basis function kernel; DNN: deep neural network.* 14

Supplementary Table 3. Coarse search to optimize the hyperparameters of the processing algorithms to classify the data based on the spectral information using the validation and training set with 2000 pixels per class. *HP: Hyperparameter; I: Initial value; S: Step value; F: Final value; γ: Lambda; C: Cost; T: Number of trees; K: Number of nearest neighbours; L: Size of the output layers; EBEAE: Extended blind end-member and abundance extraction; NEBEAE: Nonlinear extended blind end-member and abundance extraction; RF: Random forest; KNN-E: K-nearest neighbour with Euclidean distance; KNN-C: K-nearest neighbour with cosine distance; SVM-L: Support vector machine with linear kernel; SVM-RBF: Support vector machine with radial basis function kernel; DNN: deep neural network.* 15

Supplementary Table 4. Coarse search to optimize the hyperparameters of the processing algorithms to classify the data based on the spectral information using the validation and training set with 4000 pixels per class. *HP: Hyperparameter; I: Initial value; S: Step value; F: Final value; γ: Lambda; C: Cost; T: Number of trees; K: Number of nearest neighbours; L: Size of the output layers; EBEAE: Extended blind end-member and abundance extraction; NEBEAE: Nonlinear extended blind end-member and abundance extraction; RF: Random forest; KNN-E: K-nearest neighbour with Euclidean distance; KNN-C: K-nearest neighbour with cosine distance; SVM-L: Support vector machine with linear kernel; SVM-RBF: Support vector machine with radial basis function kernel; DNN: deep neural network.* 16

Supplementary Table 5. Summary of the studies found in the literature which employs intraoperative fluorescence imaging for in-vivo brain tumour detection. *5-ALA: 5-Aminolevulimic Acid. ICG: Indocyanine Green. FS: Fluorescein Sodium. n/a: Not Available.* 17

Supplementary Table 6. Detailed patient demographics and tumour characteristics. *n/a: Not Available; Opx: Operation number x; G1: Grade 1; G2: Grade 2; G3: Grade 3; G4: Grade 4.* 18

Supplementary Table 7. Data partition detail of the five folds. *Asterisk indicates patients without tumour samples labelled. Opx: Operation number* x. 19

Supplementary Table 8. Summary of the total number of labelled pixels per class and fold divided by training, validation, and test sets. *NT: Normal; TT: Tumour; BV: Blood vessel; BG: Background.* 20

Supplementary Figures

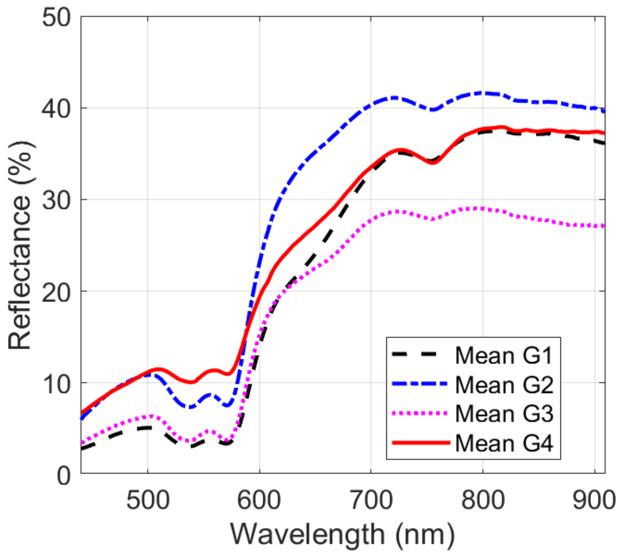

Supplementary Figure 1. Average spectral signature of the entire labelled dataset for each primary tumour grade. *G1: Grade 1; G2: Grade 2; G3: Grade 3; G4: Grade 4.*

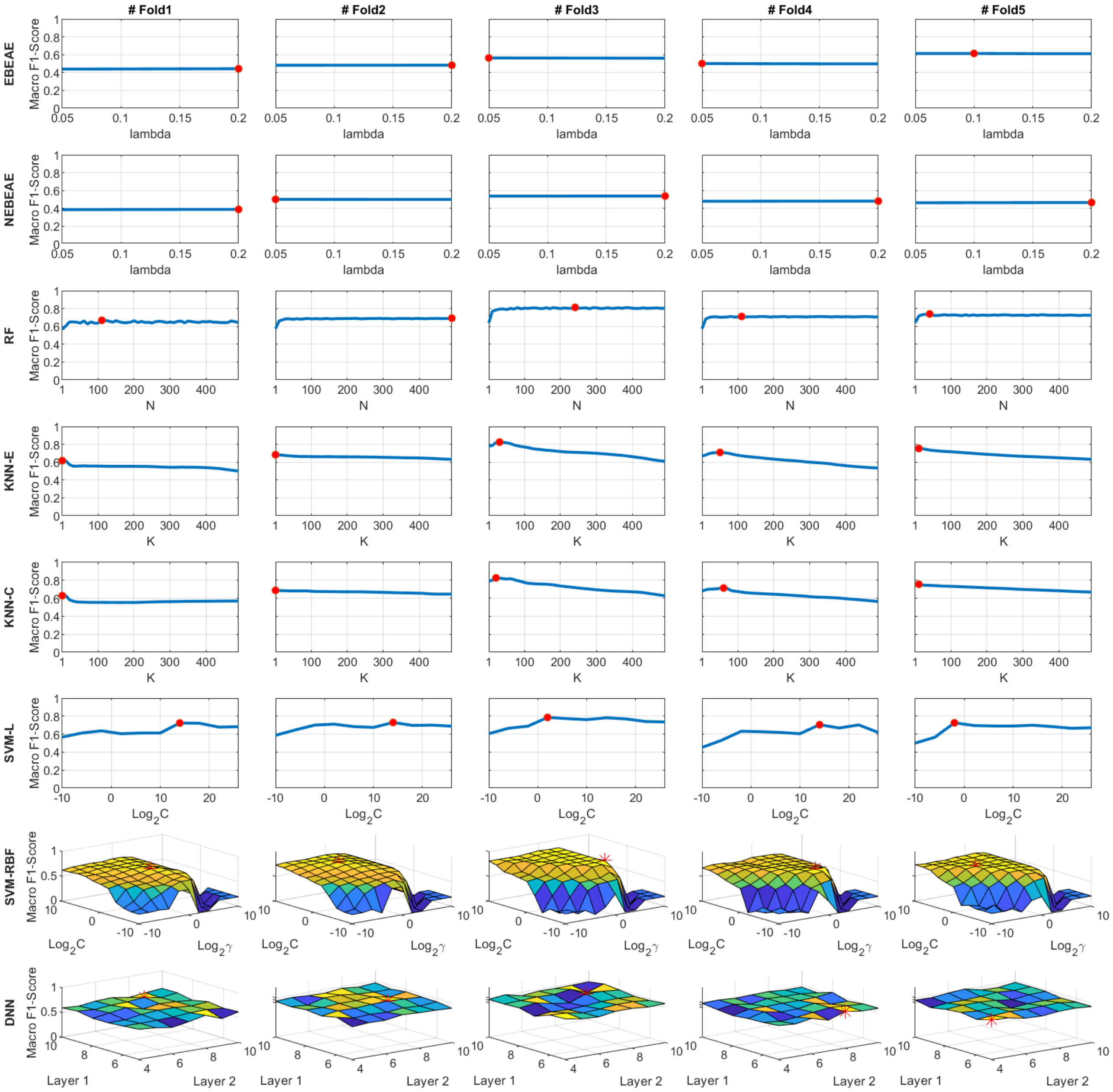

Supplementary Figure 2. Coarse search representation of the macro F1-Score results using the data reduction of 1,000 pixels per class for the hyperparameter optimization of each classification algorithm using the validation set. Red dots and asterisks represent the optimal value. *N: Number of trees; K: Number of nearest neighbours; γ: Lambda; C: Cost.*

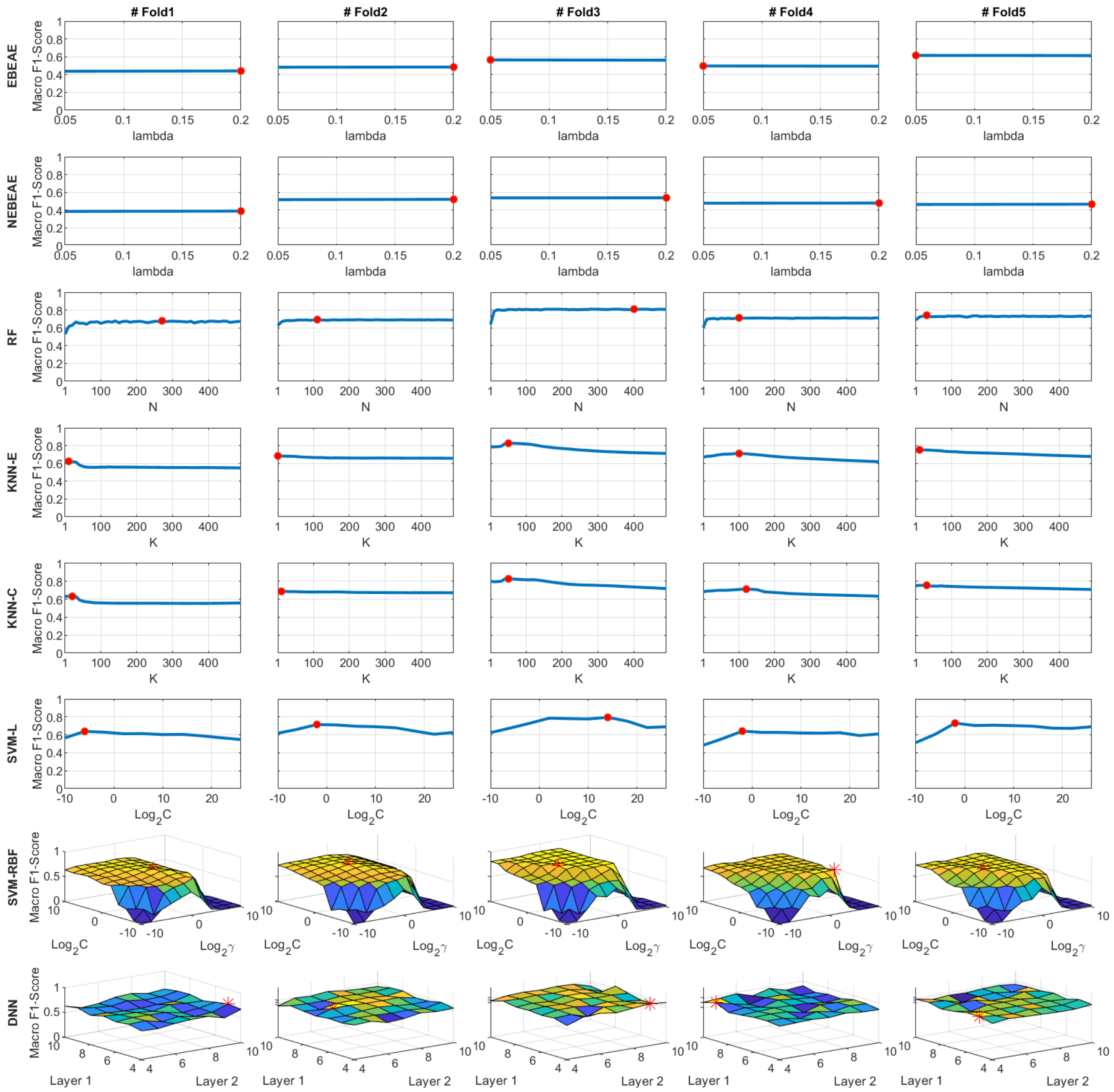

Supplementary Figure 3. Coarse search representation of the macro F1-Score results using the data reduction of 2,000 pixels per class for the hyperparameter optimization of each classification algorithm using the validation set. Red dots and asterisks represent the optimal value. *N: Number of trees; K: Number of nearest neighbours; γ: Lambda; C: Cost.*

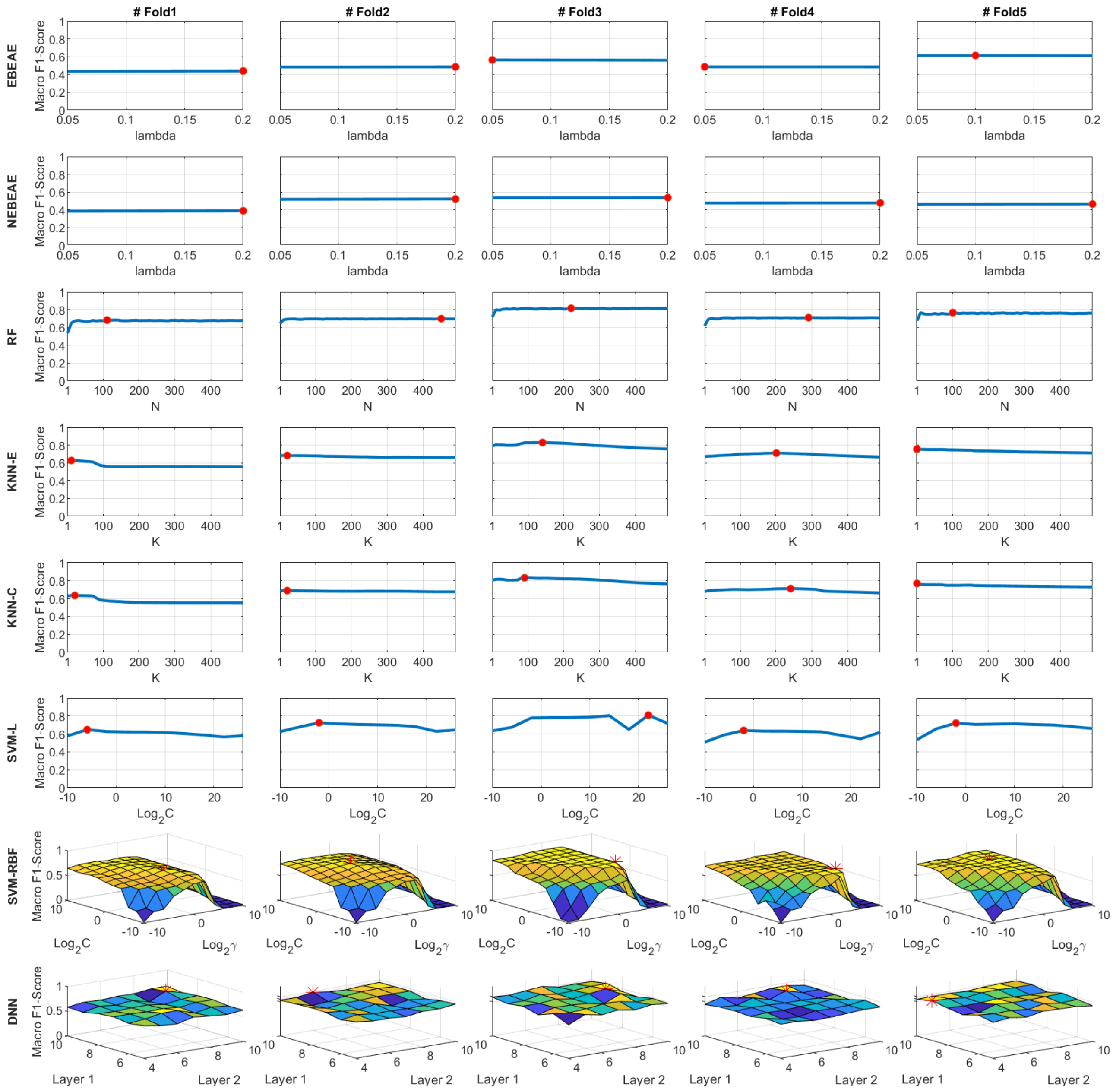

Supplementary Figure 4. Coarse search representation of the macro F1-Score results using the data reduction of 4,000 pixels per class for the hyperparameter optimization of each classification algorithm using the validation set. Red dots and asterisks represent the optimal value. *N: Number of trees; K: Number of nearest neighbours; γ: Lambda; C: Cost.*

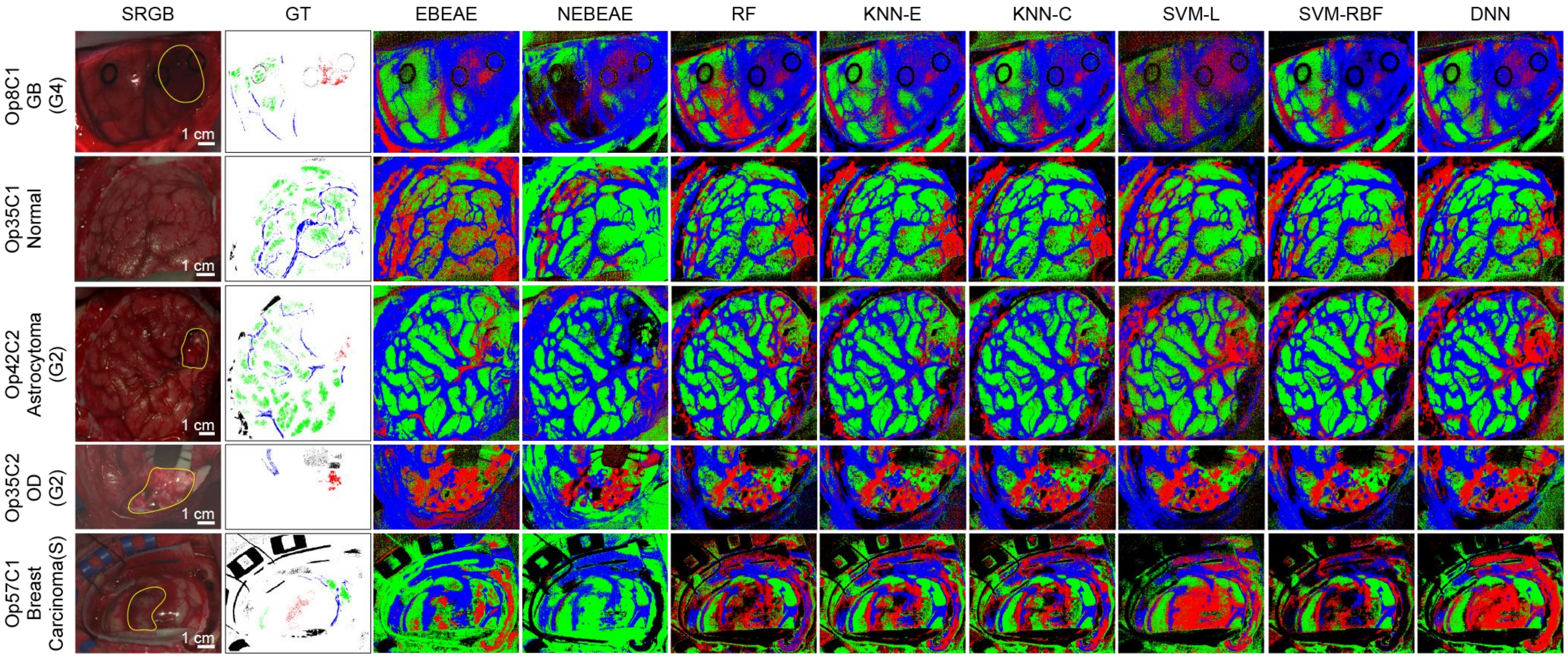

Supplementary Figure 5. Examples of synthetic RGB (SRGB) images, ground truth (GT) maps and supervised classification maps generated using the eight algorithms with the optimal hyperparameters from different tumour types of the validation set. Approximate tumour areas were surrounded in yellow line on the SRGB image by the operating surgeon according to the intraoperative neuronavigation and the definitive pathological diagnosis of the resected tissue. Rubber ring markers were employed in some cases (e.g., Op8C1) to indicate the area where the biopsies for pathology were resected. In the GT maps and classification maps, red represents tumour class, green normal class, blue blood vessel class, and black background class. *Opx: Operation number x; Cy: Capture number y*.

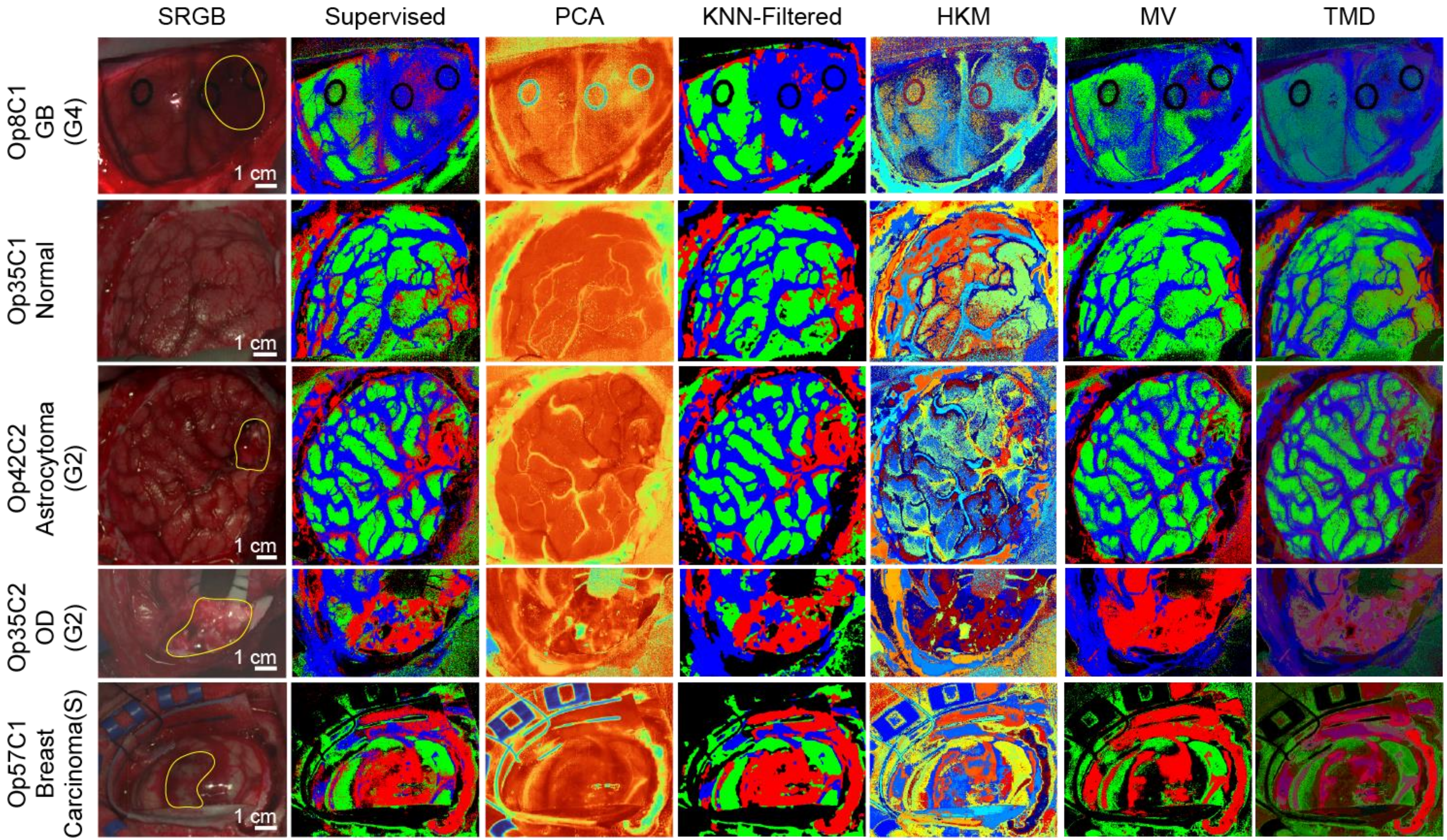

Supplementary Figure 6. Example of SRGB images and output maps from different tumour types of the validation set at the different stages of the proposed framework (based on the DNN as supervised algorithm using the optimal hyperparameters). Approximate tumour areas were surrounded in yellow line on the SRGB image by the operating surgeon according to the intraoperative neuronavigation and the definitive pathological diagnosis of the resected tissue. In the GT, supervised, KNN-filtered, and MV maps, red represents tumour class, green normal class, blue blood vessel class, and black background class. PCA maps represent the value of the first principal component (blue color represents the minimum value and red the maximum). HKM maps represent 24 different clusters and colors have no meaning. *Opx: Operation number x; Cy: Capture number y*.

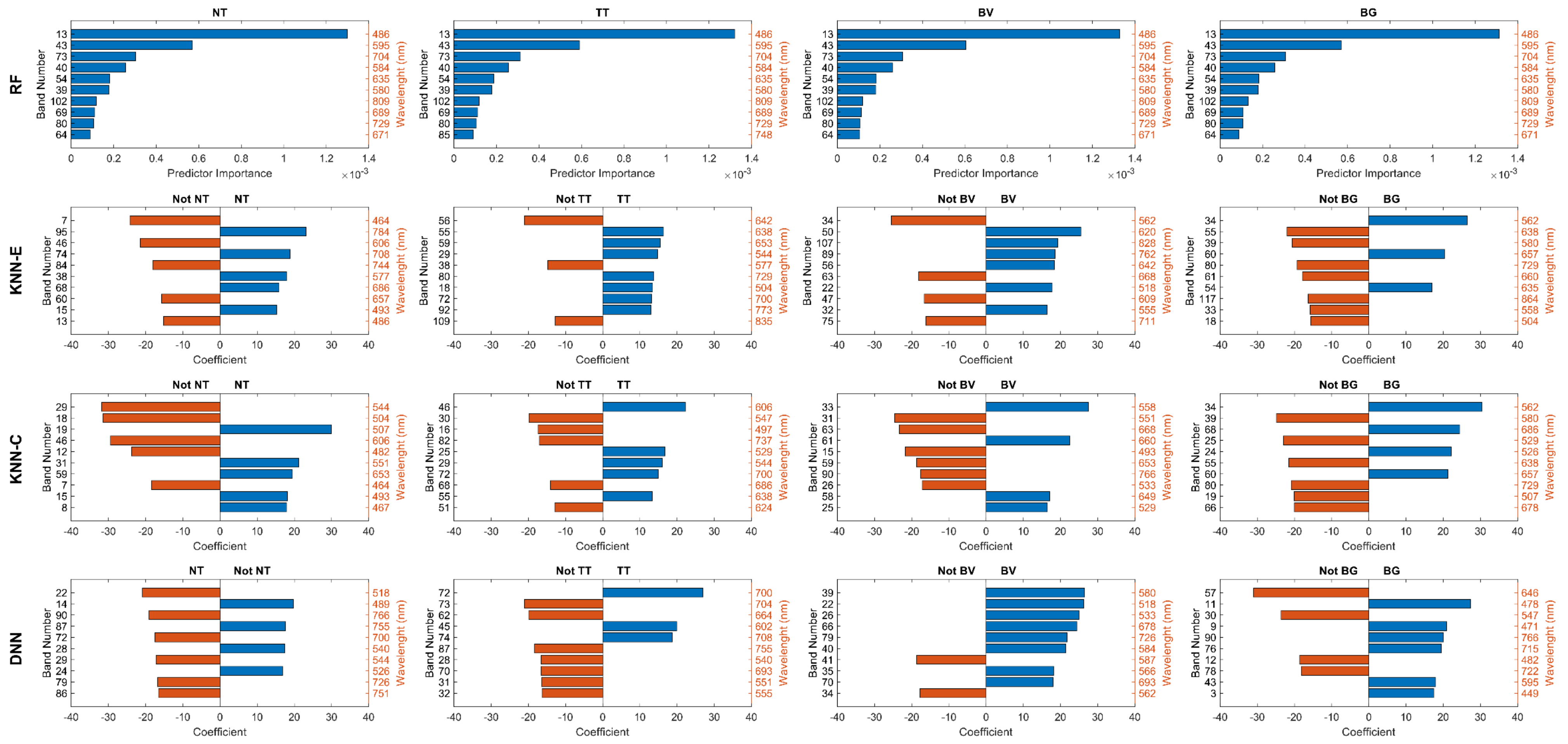

Supplementary Figure 7. Ten most relevant features identified using the LIME approach for the classification models by the RF, KNN-E, KNN-C and DNN algorithms using the training set of the first fold in each class.

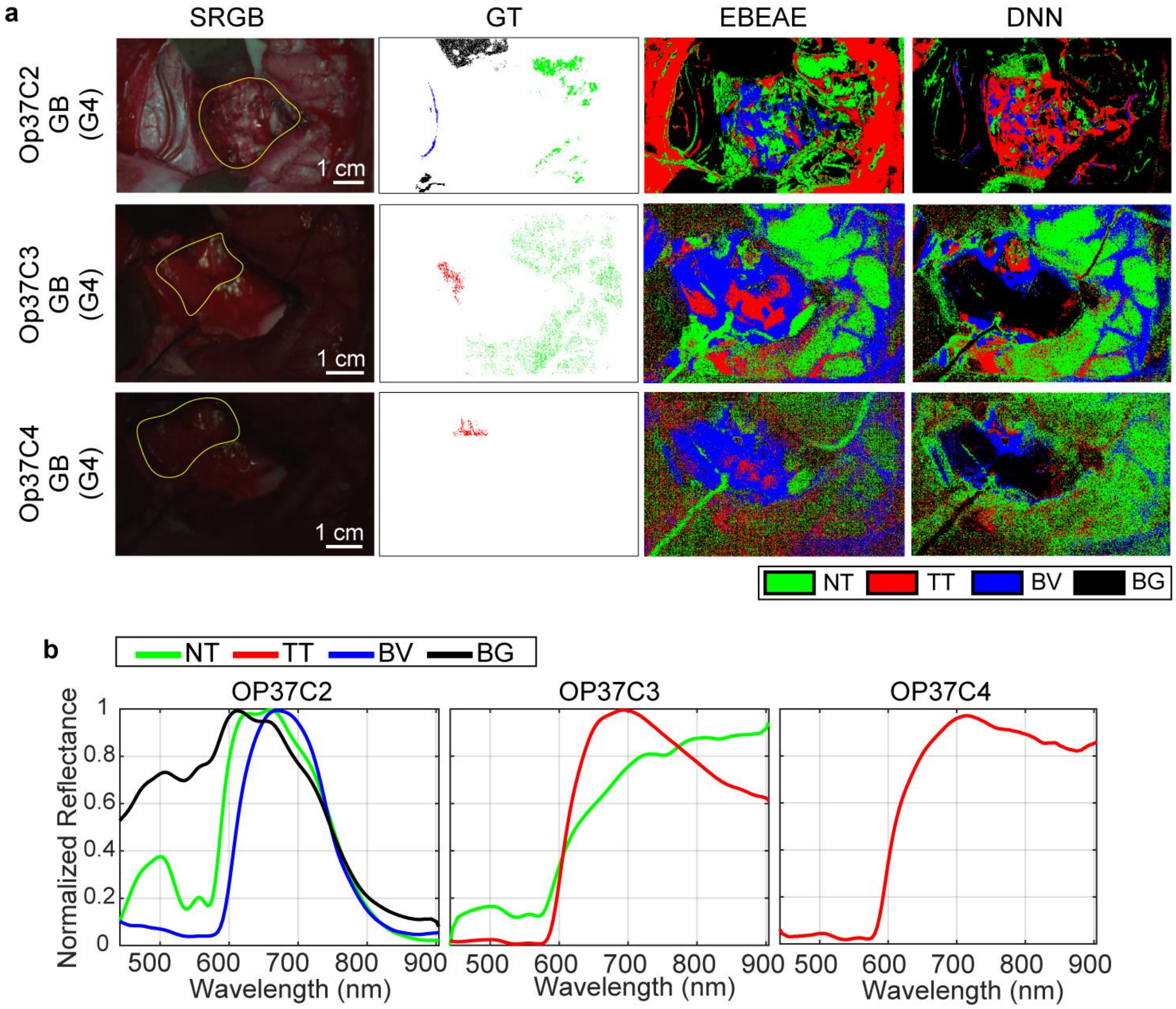

Supplementary Figure 8. Examples of the limitations related to deep-layer tumours. a, Example of synthetic RGB images, GT maps and supervised classification maps created using the EBEAE and DNN algorithms with the optimal hyperparameters from a deep-layer tumour captured in non-optimal conditions in the validation set. Approximate tumour areas were surrounded in yellow on the SRGB image by the operating surgeon according to the intraoperative neuronavigation and the definitive pathological diagnosis of the resected tissue. **b**, Average spectral signatures (solid lines) of the GT pixels from **a**. In the ground-truth map and classification maps, red represents tumour class, green normal class, blue blood vessel class, and black background class. *Opx: Operation number x; Cy: Capture number y*.

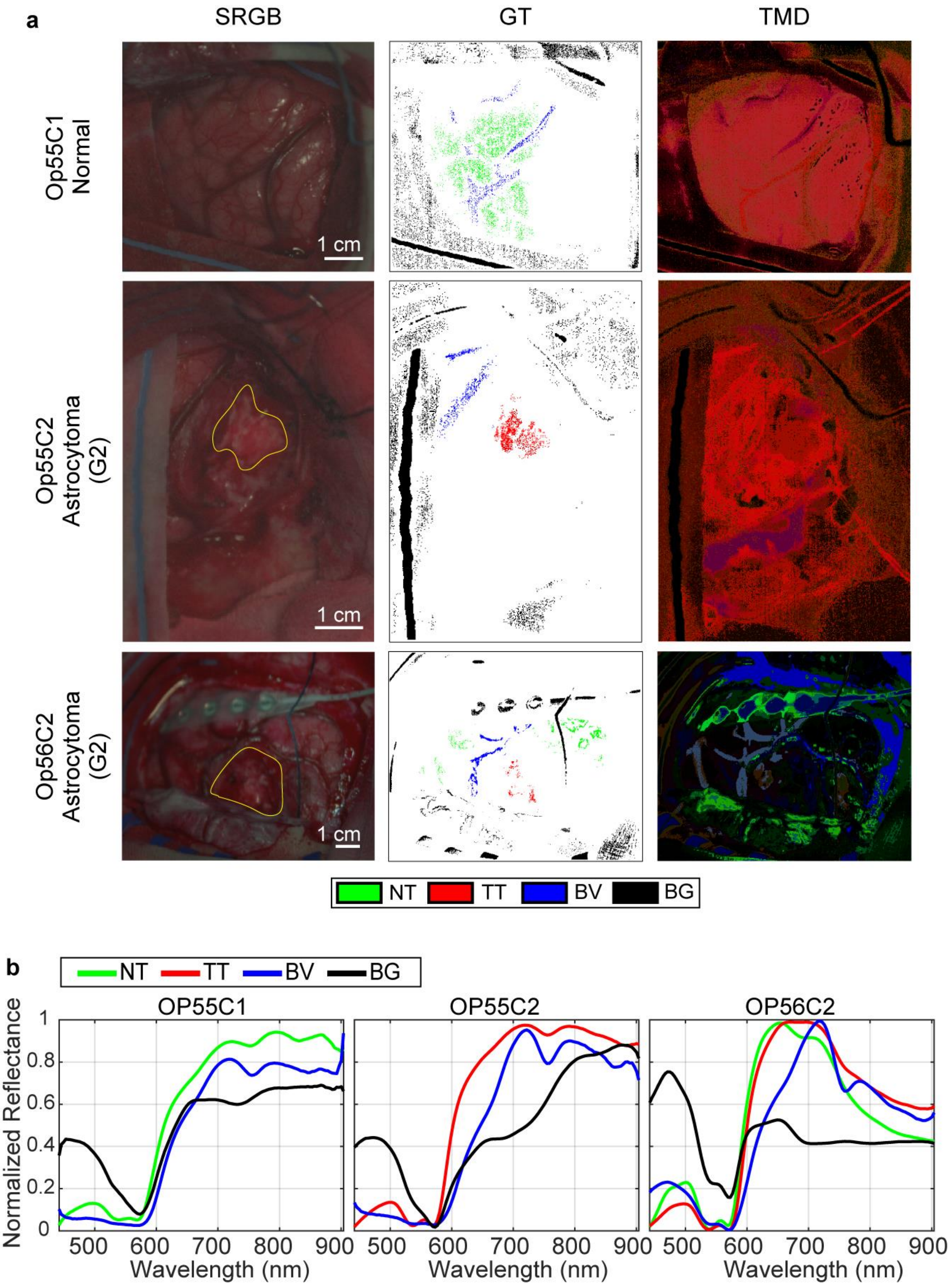

Supplementary Figure 9. Examples of the limitations of the proposed framework after processing the test set. a, Example of SRGB images, GT maps and TMD maps (based on the DNN algorithm) from HS images captured in non-optimal conditions in the test set. Approximate tumour areas were surrounded in yellow on the SRGB image by the operating surgeon according to the intraoperative neuronavigation and the definitive pathological diagnosis of the resected tissue **b**, Average spectral signatures (solid lines) of the GT pixels from **a.** *Opx: Operation number x; Cy: Capture number y*.

Supplementary Tables

Supplementary Table 1. Summary of the in-vivo HS human brain image database, including number of pixels labelled of each class, spatial and spectral dimension, and the diagnosis. *NT: Normal; TT: Tumour; BV: Blood vessel; BG: Background; S: Secondary; P: Primary; G1: Grade 1; G2: Grade 2; G3: Grade 3; G4: Grade 4; DL: Deep layer tumour. Opx: Operation number x; Cy: Capture number y.*

Campaign	Image ID	Size (height×width×bands)	#Labeled Pixels				Diagnosis
			NT	TT	BV	BG	
First Data Campaign	Op04C2	389×345×826	5,007	0	965	1,992	Normal Brain
	Op05C1	483×488×826	6,061	0	1,727	20,483	Renal Carcinoma (S)
	Op07C1	582×400×826	7,714	0	1,089	0	Normal Brain
	Op08C1	460×549×826	2,295	1,221	1,331	630	G4 Glioblastoma (P)
	Op08C2	480×553×826	2,187	138	1,000	7,444	G4 Glioblastoma (P)
	Op10C3	371×461×826	10,626	0	2,332	3,972	G4 Glioblastoma (P)
	Op12C1	443×497×826	4,516	855	8,697	1,685	G4 Glioblastoma (P)
	Op12C2	445×498×826	6,553	3,139	6,041	8,731	G4 Glioblastoma (P)
	Op13C1	298×253×826	1,827	0	129	589	Lung Carcinoma (S)
	Op14C1	317×244×826	0	30	64	1,866	G4 Glioblastoma (P)
	Op15C1	376×494×826	1,251	2,046	4,089	696	G4 Glioblastoma (P)
	Op16C1	335×323×826	3,970	0	246	12,002	Normal Brain
	Op16C2	335×326×826	349	0	0	2,767	Normal Brain
	Op16C3	315×321×826	603	0	234	1,696	Normal Brain
	Op16C4	383×297×826	1,178	0	1,064	956	G4 Glioblastoma (P)
	Op16C5	414×292×826	2,643	0	452	5,125	G4 Glioblastoma (P)
	Op17C1	441×399×826	1,328	0	68	3,069	G4 Glioblastoma (P)
	Op18C1	479×462×826	13,450	0	488	9,773	G1 Ganglioglioma (P)
	Op18C2	510×434×826	4,813	0	958	5,895	G1 Ganglioglioma (P)
	Op19C1	601×535×826	6,499	0	1,350	1,933	G1 Meningioma (P)
	Op20C1	378×330×826	1,842	3,655	1,513	2,625	G4 Glioblastoma (P)
	Op21C1	452×334×826	3,405	167	793	5,330	Breast Carcinoma (S)
	Op21C2	448×324×826	2,353	31	555	2,137	Breast Carcinoma (S)
	Op21C5	433×340×826	969	0	1,637	1,393	Breast Carcinoma (S)
	Op22C1	597×527×826	2,806	0	1,064	3,677	G3 Anaplastic Oligodendroglioma (P)
	Op22C2	611×527×826	8,174	0	680	0	G3 Anaplastic Oligodendroglioma (P)
	Op22C3	592×471×826	0	96	0	0	G3 Anaplastic Oligodendroglioma (P)
Second Data Campaign	Op34C1	319×356×826	0	0	0	15,609	G3 Anaplastic Astrocytoma (P)
	Op34C2	300×342×826	512	145	0	12,979	G3 Anaplastic Astrocytoma (P)
	Op34C3	290×301×826	0	360	0	10,533	G3 Anaplastic Astrocytoma (P)
	Op35C1	431×503×826	9,025	0	7,287	485	G2 Oligodendroglioma (P) [DL]
	Op35C2	312×535×826	0	1,338	629	1,353	G2 Oligodendroglioma (P)
	Op36C1	412×324×826	11,665	0	4,461	4,621	G4 Glioblastoma (P) [DL]
	Op36C2	432×322×826	2,940	888	2,980	5,853	G4 Glioblastoma (P)
	Op37C1	434×453×826	12,719	0	2,524	11,161	G4 Glioblastoma (P) [DL]
	Op37C2	315×526×826	2,997	0	375	4,166	G4 Glioblastoma (P)
	Op37C3	290×422×826	3,201	407	0	0	G4 Glioblastoma (P)
	Op37C4	280×444×826	0	330	0	0	G4 Glioblastoma (P)
	Op38C1	497×490×826	18,511	2,295	4,229	3,669	G1 Meningioma (P)
	Op39C1	415×446×826	4,003	244	489	9,829	G4 Glioblastoma (P)
	Op39C2	399×439×826	7,705	1,629	822	9,867	G4 Glioblastoma (P)
	Op40C1	303×374×826	2,728	394	1,151	3,492	G1 Meningioma (P)
	Op40C2	294×344×826	817	700	2,130	902	G1 Meningioma (P)
	Op41C1	449×486×826	2,359	69	1,047	5,030	G1 Ganglioglioma (P)
	Op41C2	437×488×826	4,874	158	2,150	4,888	G1 Ganglioglioma (P)
	Op42C1	629×646×826	20,565	0	10,956	8,991	G2 Astrocytoma (glioma) (P)
	Op42C2	623×584×826	19,435	428	5,110	3,983	G2 Astrocytoma (glioma) (P)
	Op42C3	650×582×826	2,385	401	979	716	G2 Astrocytoma (glioma) (P)
	Op43C1	575×543×826	28,285	1,177	4,012	5,995	G4 Glioblastoma (P)
	Op43C2	554×446×826	17,236	475	2,103	1,964	G4 Glioblastoma (P)
	Op43C4	538×525×826	14,160	0	4,749	687	G4 Glioblastoma (P)
Third Data Campaign	Op50C1	565×533×826	2,116	1,091	620	5,502	G1 Meningioma (P)
	Op51C1	635×617×826	1,164	0	424	31,247	G4 Glioblastoma (P)
	Op53C1	546×446×826	361	5,549	0	33,606	Breast Carcinoma (S)
	Op54C1	515×504×826	2,697	0	3,506	9,535	G4 Glioblastoma (P)
	Op55C1	397×435×826	3,128	0	901	8,278	G3 Astrocytoma (glioma) (P)
	Op55C2	500×349×826	0	1,046	545	9,415	G3 Astrocytoma (glioma) (P)
	Op56C1	446×598×826	1,346	4,081	2,200	28,370	G2 Astrocytoma (glioma) (P)
	Op56C2	467×566×826	1,326	372	1,116	7,702	G2 Astrocytoma (glioma) (P)
	Op57C1	440×535×826	1,773	771	1,263	23,415	Breast Carcinoma (S)
	Op58C2	721×752×826	6,589	1,629	4,565	43,565	G2 Meningioma (P)
**Total number of labelled pixels**			**309,041**	**37,355**	**111,889**	**433,874**	

Supplementary Table 2. Coarse search to optimize the hyperparameters of the processing algorithms to classify the data based on the spectral information using the validation and training set with 1000 pixels per class. *HP: Hyperparameter; I: Initial value; S: Step value; F: Final value; γ: Lambda; C: Cost; T: Number of trees; K: Number of nearest neighbours; L: Size of the output layers; EBEAE: Extended blind end-member and abundance extraction; NEBEAE: Nonlinear extended blind end-member and abundance extraction; RF: Random forest; KNN-E: K-nearest neighbour with Euclidean distance; KNN-C: K-nearest neighbour with cosine distance; SVM-L: Support vector machine with linear kernel; SVM-RBF: Support vector machine with radial basis function kernel; DNN: deep neural network.*

Classifier	HP	# Fold	Coarse Search		Macro F1-Score (%)
			I/S/F	Optimal	
EBEAE	γ	1	0.05/0.05/0.2	0.20	0.443
		2	0.05/0.05/0.2	0.20	0.482
		3	0.05/0.05/0.2	0.05	0.564
		4	0.05/0.05/0.2	0.05	0.501
		5	0.05/0.05/0.2	0.10	0.614
NEBEAE	γ	1	0.05/0.05/0.2	0.20	0.389
		2	0.05/0.05/0.2	0.05	0.501
		3	0.05/0.05/0.2	0.20	0.538
		4	0.05/0.05/0.2	0.20	0.482
		5	0.05/0.05/0.2	0.20	0.466
RF	T	1	1/10/500	50	0.668
		2	1/10/500	50	0.692
		3	1/10/500	50	0.813
		4	1/10/500	50	0.711
		5	1/10/500	50	0.740
KNN-E	N	1	1/10/500	1	0.617
		2	1/10/500	1	0.684
		3	1/10/500	31	0.825
		4	1/10/500	51	0.710
		5	1/10/500	11	0.754
KNN-C	N	1	1/10/500	1	0.630
		2	1/10/500	1	0.688
		3	1/10/500	21	0.828
		4	1/10/500	61	0.714
		5	1/10/500	11	0.756
SVM-L	C	1	$2^{-10}/2^{4}/2^{26}$	2^{14}	0.724
		2	$2^{-10}/2^{4}/2^{26}$	2^{14}	0.730
		3	$2^{-10}/2^{4}/2^{26}$	2^{2}	0.786
		4	$2^{-10}/2^{4}/2^{26}$	2^{14}	0.705
		5	$2^{-10}/2^{4}/2^{26}$	2^{-2}	0.726
SVM-RBF	C<br>γ	1	$2^{-10}/2^{2}/2^{10}$<br>$2^{-10}/2^{2}/2^{10}$	2^{0}<br>2^{0}	0.711
		2	$2^{-10}/2^{2}/2^{10}$<br>$2^{-10}/2^{2}/2^{10}$	2^{4}<br>2^{-2}	0.787
		3	$2^{-10}/2^{2}/2^{10}$<br>$2^{-10}/2^{2}/2^{10}$	2^{-2}<br>2^{4}	0.852
		4	$2^{-10}/2^{2}/2^{10}$<br>$2^{-10}/2^{2}/2^{10}$	2^{-4}<br>2^{2}	0.762
		5	$2^{-10}/2^{2}/2^{10}$<br>$2^{-10}/2^{2}/2^{10}$	2^{2}<br>2^{-4}	0.784
DNN	L 1<br>L 2	1	4/1/10<br>4/1/10	9<br>8	0.670
		2	4/1/10<br>4/1/10	7<br>8	0.776
		3	4/1/10<br>4/1/10	8<br>8	0.830
		4	4/1/10<br>4/1/10	5<br>8	0.747
		5	4/1/10<br>4/1/10	5<br>4	0.807

Supplementary Table 3. Coarse search to optimize the hyperparameters of the processing algorithms to classify the data based on the spectral information using the validation and training set with 2000 pixels per class. *HP: Hyperparameter; I: Initial value; S: Step value; F: Final value; γ: Lambda; C: Cost; T: Number of trees; K: Number of nearest neighbours; L: Size of the output layers; EBEAE: Extended blind end-member and abundance extraction; NEBEAE: Nonlinear extended blind end-member and abundance extraction; RF: Random forest; KNN-E: K-nearest neighbour with Euclidean distance; KNN-C: K-nearest neighbour with cosine distance; SVM-L: Support vector machine with linear kernel; SVM-RBF: Support vector machine with radial basis function kernel; DNN: deep neural network.*

Classifier	HP	# Fold	Coarse Search		Macro F1-Score (%)
			I/S/F	Optimal	
EBEAE	γ	1	0.05/0.05/0.2	0.20	0.441
		2	0.05/0.05/0.2	0.20	0.484
		3	0.05/0.05/0.2	0.05	0.565
		4	0.05/0.05/0.2	0.05	0.497
		5	0.05/0.05/0.2	0.05	0.615
NEBEAE	γ	1	0.05/0.05/0.2	0.20	0.389
		2	0.05/0.05/0.2	0.20	0.521
		3	0.05/0.05/0.2	0.20	0.538
		4	0.05/0.05/0.2	0.20	0.479
		5	0.05/0.05/0.2	0.20	0.466
RF	T	1	1/10/500	50	0.680
		2	1/10/500	50	0.693
		3	1/10/500	50	0.811
		4	1/10/500	50	0.713
		5	1/10/500	50	0.742
KNN-E	N	1	1/10/500	11	0.624
		2	1/10/500	1	0.685
		3	1/10/500	51	0.826
		4	1/10/500	101	0.711
		5	1/10/500	11	0.753
KNN-C	N	1	1/10/500	21	0.633
		2	1/10/500	11	0.688
		3	1/10/500	51	0.828
		4	1/10/500	121	0.713
		5	1/10/500	31	0.756
SVM-L	C	1	$2^{-10}/2^{4}/2^{26}$	2^{-6}	0.639
		2	$2^{-10}/2^{4}/2^{26}$	2^{-2}	0.715
		3	$2^{-10}/2^{4}/2^{26}$	2^{14}	0.795
		4	$2^{-10}/2^{4}/2^{26}$	2^{-2}	0.641
		5	$2^{-10}/2^{4}/2^{26}$	2^{-2}	0.730
SVM-RBF	C<br>γ	1	$2^{-10}/2^{2}/2^{10}$<br>$2^{-10}/2^{2}/2^{10}$	2^{2}<br>2^{-2}	0.937
		2	$2^{-10}/2^{2}/2^{10}$<br>$2^{-10}/2^{2}/2^{10}$	2^{6}<br>2^{-6}	0.820
		3	$2^{-10}/2^{2}/2^{10}$<br>$2^{-10}/2^{2}/2^{10}$	2^{4}<br>2^{-2}	0.896
		4	$2^{-10}/2^{2}/2^{10}$<br>$2^{-10}/2^{2}/2^{10}$	2^{0}<br>2^{0}	0.897
		5	$2^{-10}/2^{2}/2^{10}$<br>$2^{-10}/2^{2}/2^{10}$	2^{0}<br>2^{0}	0.899
DNN	L 1<br>L 2	1	4/1/10<br>4/1/10	5<br>10	0.707
		2	4/1/10<br>4/1/10	7<br>5	0.759
		3	4/1/10<br>4/1/10	4<br>9	0.844
		4	4/1/10<br>4/1/10	9<br>4	0.779
		5	4/1/10<br>4/1/10	5<br>4	0.805

Supplementary Table 4. Coarse search to optimize the hyperparameters of the processing algorithms to classify the data based on the spectral information using the validation and training set with 4000 pixels per class. *HP: Hyperparameter; I: Initial value; S: Step value; F: Final value; γ: Lambda; C: Cost; T: Number of trees; K: Number of nearest neighbours; L: Size of the output layers; EBEAE: Extended blind end-member and abundance extraction; NEBEAE: Nonlinear extended blind end-member and abundance extraction; RF: Random forest; KNN-E: K-nearest neighbour with Euclidean distance; KNN-C: K-nearest neighbour with cosine distance; SVM-L: Support vector machine with linear kernel; SVM-RBF: Support vector machine with radial basis function kernel; DNN: deep neural network.*

**Classifier**	**HP**	**# Fold**	**Coarse Search**		**Macro F1-Score**
			**I/S/F**	**Optimal**	**(%)**
EBEAE	γ	1	0.05/0.05/0.2	0.20	0.440
		2	0.05/0.05/0.2	0.20	0.485
		3	0.05/0.05/0.2	0.05	0.564
		4	0.05/0.05/0.2	0.05	0.486
		5	0.05/0.05/0.2	0.10	0.614
NEBEAE	γ	1	0.05/0.05/0.2	0.20	0.389
		2	0.05/0.05/0.2	0.20	0.523
		3	0.05/0.05/0.2	0.20	0.537
		4	0.05/0.05/0.2	0.20	0.478
		5	0.05/0.05/0.2	0.20	0.465
RF	T	1	1/10/500	50	0.684
		2	1/10/500	50	0.700
		3	1/10/500	50	0.816
		4	1/10/500	50	0.711
		5	1/10/500	50	0.767
KNN-E	N	1	1/10/500	11	0.627
		2	1/10/500	21	0.683
		3	1/10/500	141	0.829
		4	1/10/500	201	0.711
		5	1/10/500	1	0.755
KNN-C	N	1	1/10/500	21	0.635
		2	1/10/500	21	0.689
		3	1/10/500	91	0.832
		4	1/10/500	241	0.711
		5	1/10/500	1	0.769
SVM-L	C	1	$2^{-10}/2^{4}/2^{26}$	2^{-6}	0.648
		2	$2^{-10}/2^{4}/2^{26}$	2^{-2}	0.726
		3	$2^{-10}/2^{4}/2^{26}$	2^{22}	0.810
		4	$2^{-10}/2^{4}/2^{26}$	2^{-2}	0.639
		5	$2^{-10}/2^{4}/2^{26}$	2^{-2}	0.722
SVM-RBF	C<br>γ	1	$2^{-10}/2^{2}/2^{10}$<br>$2^{-10}/2^{2}/2^{10}$	2^{-2}<br>2^{0}	0.715
		2	$2^{-10}/2^{2}/2^{10}$<br>$2^{-10}/2^{2}/2^{10}$	2^{2}<br>2^{-2}	0.795
		3	$2^{-10}/2^{2}/2^{10}$<br>$2^{-10}/2^{2}/2^{10}$	2^{-4}<br>2^{4}	0.854
		4	$2^{-10}/2^{2}/2^{10}$<br>$2^{-10}/2^{2}/2^{10}$	2^{-6}<br>2^{4}	0.767
		5	$2^{-10}/2^{2}/2^{10}$<br>$2^{-10}/2^{2}/2^{10}$	2^{4}<br>2^{0}	0.792
DNN	L 1<br>L 2	1	4/1/10<br>4/1/10	9<br>9	0.717
		2	4/1/10<br>4/1/10	10<br>6	0.775
		3	4/1/10<br>4/1/10	8<br>9	0.851
		4	4/1/10<br>4/1/10	9<br>8	0.784
		5	4/1/10<br>4/1/10	9<br>4	0.807

Supplementary Table 5. Summary of the studies found in the literature which employs intraoperative fluorescence imaging for in-vivo brain tumour detection. *5-ALA: 5-Aminolevulimic Acid. ICG: Indocyanine Green. FS: Fluorescein Sodium. n/a: Not Available.*

Reference	Year	Contrast Agent	Excitation (nm)	Emission (nm)	#Patients	#Biopsies	Tumour Grade	Tumour Sensitivity (%)
Valdes *et al.*[1]	2015	5-ALA	375 – 440	640 – 710	12	73	1 and 2	58.3
Lee *et al.*[2]	2017	ICG	778	700 – 850	14	46	1 and 2	96.4
Acerbi *et al.*[3]	2018	FS	460–500	540–690	13	50	3 and 4	80.8
Molina *et al.*[4]	2020	5-ALA	375 – 440	640 – 710	32	128	3 and 4	70.8
Cho *et al.*[5]	2020	ICG	778	700 – 850	36	78	4	97.0
Sweeney *et al.*[6]	2022	FS	460–500	540–690	98	n/a	4	62.0

Supplementary Table 6. Detailed patient demographics and tumour characteristics. *n/a: Not Available; Opx: Operation number x; G1: Grade 1; G2: Grade 2; G3: Grade 3; G4: Grade 4.*

ID	Age	Sex	Tumour Location	Tumour Size (mm)	Histologic Type	Tumour Type	Grade/Origin
Op4	59	Female	Right Frontal Lobe	26×32×30	Metastasis (Lung Adenocarcinoma)	Secondary	Lung
Op5	50	Male	Right Parietal Lobe	32×29×28	Metastasis (Renal Carcinoma)	Secondary	Kidney
Op7	63	Male	Right Temporal Lobe	47×31×34	Glioblastoma	Primary	G4
Op8	71	Male	Left Parietal Lobe	60×52×30	Glioblastoma	Primary	G4
Op10	68	Female	Left Frontal Lobe	35×30×30	Glioblastoma	Primary	G4
Op12	60	Male	Right Occipital Lobe	59×41×42	Glioblastoma	Primary	G4
Op13	47	Male	Right Temporal Lobe	42×32×30	Metastasis (Lung Carcinoma)	Secondary	Lung
Op14	62	Female	Right Parietal Lobe	30×19×26	Glioblastoma	Primary	G4
Op15	40	Male	Right Occipital Lobe	52×34×30	Glioblastoma	Primary	G4
Op16	73	Male	Right Frontal Lobe	61×29×65	Glioblastoma	Primary	G4
Op17	69	Male	Left Frontal Lobe	64×50×53	Glioblastoma	Primary	G4
Op18	30	Male	Left Frontal Lobe	39×39×42	Ganglioglioma	Primary	G1
Op19	62	Male	Right Temporal Lobe	44×39×32	Meningioma	Primary	G2
Op20	56	Male	Left Parietal Lobe	22×18×18	Glioblastoma	Primary	G4
Op21	57	Female	Left Parietal Lobe	30×31×30	Metastasis (Breast Carcinoma)	Secondary	Breast
Op22	56	Male	Left Frontal Lobe	17×10×14	Anaplastic Oligodendroglioma	Primary	G3
Op34	66	Male	Left Parietal Lobe	33×10×35	Anaplastic Astrocytoma	Primary	G3
Op35	46	Male	Left Frontal Lobe	75×45×68	Oligodendroglioma	Primary	G2
Op36	51	Female	Right Temporal Lobe	30×33×35	Glioblastoma	Primary	G4
Op37	58	Male	Left Occipital Lobe	51×31×37	Glioblastoma	Primary	G4
Op38	44	Female	Right Temporal Lobe	n/a	Meningioma	Primary	G1
Op39	70	Female	Right Parietal Lobe	43×41×53	Glioblastoma	Primary	G4
Op40	63	Male	Right Parietal Lobe	30×40×30	Meningioma	Primary	G1
Op41	n/a	Female	Right Temporal Lobe	33×22×21	Ganglioglioma	Primary	G1
Op42	66	Female	Right Temporal Lobe	29×56×39	Astrocytoma	Primary	G2
Op43	38	Male	Left Temporal Lobe	59×44×49	Glioblastoma	Primary	G4
Op50	59	Female	Right Parietal Lobe	53×34×42	Meningioma	Primary	G1
Op51	57	Male	Left Frontal Lobe	60×41×28	Glioblastoma	Primary	G4
Op53	59	Female	Cerebellum	43×37×30	Metastasis (Breast Carcinoma)	Secondary	Breast
Op54	71	Female	Right Temporal Lobe	65×50×43	Glioblastoma	Primary	G4
Op55	38	Male	Right Frontal Lobe	35×20×18	Astrocytoma (glioma)	Primary	G3
Op56	34	Male	Left Frontal Lobe	42×24×25	Astrocytoma (glioma)	Primary	G2
Op57	60	Female	Right Parietal Lobe	26×26×27	Metastasis (Breast Carcinoma)	Secondary	Breast
Op58	69	Male	Right Parietal Lobe	80×60×30	Meningioma	Primary	G2

Supplementary Table 7. Data partition detail of the five folds. *Asterisk indicates patients without tumour samples labelled. Opx: Operation number* x.

#Fold	Training Patients (#Total Images)	Validation Patients (#Total Images)	Test Patients (#Total Images)
1	Remaining 23 patients (34)	Op4*, Op8, Op22, Op39, Op41 (10)	Op10*, Op16*, Op21, Op37, Op42, Op54* (17)
2	Remaining 22 patients (37)	Op37, O41, Op42, Op50, Op58 (11)	Op4*, Op12, Op17*, Op22, Op38, Op43, Op55 (13)
3	Remaining 22 patients (41)	Op4*, Op36, Op43, Op53, Op57 (8)	Op5*, Op13*, Op18*, Op34, Op39, Op50, Op56 (12)
4	Remaining 22 patients (45)	Op10, Op18, Op36, Op50, Op54 (7)	Op7*, Op14, Op19*, Op35, Op40, Op51*, Op57 (9)
5	Remaining 22 patients (41)	Op10*, Op35, Op37, Op 40, Op50 (10)	Op8, Op15, Op20, Op36, Op41, Op53, Op58 (10)

Supplementary Table 8. Summary of the total number of labelled pixels per class and fold divided by training, validation, and test sets. *NT: Normal; TT: Tumour; BV: Blood vessel; BG: Background.*

#Fold	# Labelled Pixels				
	**NT**	**TT**	**BV**	**BG**	**Total**
**Training Set**					
**1**	179,536	32,006	71,578	316,587	599,707
**2**	122,166	23,729	48,459	305,504	499,858
**3**	184,524	20,543	81,757	227,404	514,228
**4**	231,083	32,143	80,107	332,824	676,157
**5**	228,518	17,712	70,655	302,677	619,562
**Validation Set**					
**1**	39,410	3,555	9,548	43,357	95,870
**2**	77,240	4,513	28,326	88,002	198,081
**3**	81,427	8,860	20,533	78,133	188,953
**4**	48,307	1,979	15,345	45,151	110,782
**5**	44,229	4,260	17,048	31,033	96,570
**Test Set**					
**1**	90,164	1,764	29,713	82,724	204,365
**2**	109,704	9,083	34,054	49,162	202,003
**3**	43,159	7,922	8,549	137,131	196,761
**4**	29,720	3,203	15,387	64,693	113,003
**5**	36,363	15,353	23,136	108,958	183,810

Supplementary References

1. Valdés, P. A. *et al.* Quantitative fluorescence using 5-aminolevulinic acid-induced protoporphyrin IX biomarker as a surgical adjunct in low-grade glioma surgery. *J. Neurosurg.* **123**, 771–780 (2015).
2. Lee, J. Y. K. *et al.* Near-infrared fluorescent image-guided surgery for intracranial meningioma. *J. Neurosurg.* **128**, 380–390 (2018).
3. Acerbi, F. *et al.* Fluorescein-Guided Surgery for Resection of High-Grade Gliomas: A Multicentric Prospective Phase II Study (FLUOGLIO). *Clin. Cancer Res.* **24**, 52–61 (2018).
4. Suero Molina, E., Stögbauer, L., Jeibmann, A., Warneke, N. & Stummer, W. Validating a new generation filter system for visualizing 5-ALA-induced PpIX fluorescence in malignant glioma surgery: a proof of principle study. *Acta Neurochir. (Wien).* **162**, 785–793 (2020).
5. Cho, S. S. *et al.* Near-Infrared Imaging with Second-Window Indocyanine Green in Newly Diagnosed High-Grade Gliomas Predicts Gadolinium Enhancement on Postoperative Magnetic Resonance Imaging. *Mol. Imaging Biol.* **22**, 1427–1437 (2020).
6. Sweeney, J. F. *et al.* Comparison of sodium fluorescein and intraoperative ultrasonography in brain tumor resection. *J. Clin. Neurosci.* **106**, 141–144 (2022).
