# Supplementary material for: Hyperspectral imaging benchmark based on machine learning for intraoperative brain tumour detection
Source: NPJ Precis Oncol. 2023 Nov 14;7:119. doi: 10.1038/s41698-023-00475-9 (PMC10646050; doi:10.1038/s41698-023-00475-9)
Supplement: Supplementary file 2 — REPORTING SUMMARY [file 41698_2023_475_MOESM2_ESM.pdf]

nature portfolio

Corresponding author(s): Himar Fabelo

Last updated by author(s): Sep 18, 2023

Reporting Summary

Nature Portfolio wishes to improve the reproducibility of the work that we publish. This form provides structure for consistency and transparency in reporting. For further information on Nature Portfolio policies, see our Editorial Policies and the Editorial Policy Checklist.

Statistics

For all statistical analyses, confirm that the following items are present in the figure legend, table legend, main text, or Methods section.

n/a	Confirmed	
☐	☒	The exact sample size (*n*) for each experimental group/condition, given as a discrete number and unit of measurement
☐	☒	A statement on whether measurements were taken from distinct samples or whether the same sample was measured repeatedly
☐	☒	The statistical test(s) used AND whether they are one- or two-sided<br>*Only common tests should be described solely by name; describe more complex techniques in the Methods section.*
☒	☐	A description of all covariates tested
☒	☐	A description of any assumptions or corrections, such as tests of normality and adjustment for multiple comparisons
☒	☐	A full description of the statistical parameters including central tendency (e.g. means) or other basic estimates (e.g. regression coefficient) AND variation (e.g. standard deviation) or associated estimates of uncertainty (e.g. confidence intervals)
☒	☐	For null hypothesis testing, the test statistic (e.g. *F*, *t*, *r*) with confidence intervals, effect sizes, degrees of freedom and *P* value noted<br>*Give P values as exact values whenever suitable.*
☒	☐	For Bayesian analysis, information on the choice of priors and Markov chain Monte Carlo settings
☒	☐	For hierarchical and complex designs, identification of the appropriate level for tests and full reporting of outcomes
☒	☐	Estimates of effect sizes (e.g. Cohen's *d*, Pearson's *r*), indicating how they were calculated

Our web collection on statistics for biologists contains articles on many of the points above.

Software and code

Policy information about availability of computer code

Data collection	For HS data collection we employed a custom acquisition sofware based on XCLIB Library (EPIX, Inc., IL, USA) and Xeneth SDK (Xenics USA, Inc.)
Data analysis	MATLAB (R2021b) was used for data analysis. Particularly, the Statistics and Machine Learning Toolbox version 12.2 and Deep Learning Toolbox version 14.3 of MATLAB were used for the machine learning development, while LIBSVM 3.22 library was used for the SVM implementation in MATLAB.

For manuscripts utilizing custom algorithms or software that are central to the research but not yet described in published literature, software must be made available to editors and reviewers. We strongly encourage code deposition in a community repository (e.g. GitHub). See the Nature Portfolio guidelines for submitting code & software for further information.

Data

Policy information about availability of data

All manuscripts must include a data availability statement. This statement should provide the following information, where applicable:

- Accession codes, unique identifiers, or web links for publicly available datasets
- A description of any restrictions on data availability
- For clinical datasets or third party data, please ensure that the statement adheres to our policy

The authors declare that all data supporting the results of this study are available within the paper and its Supplementary Information. The datasets generated during the current study are available, under reasonable request, through https://hsibraindatabase.iuma.ulpgc.es/.

Research involving human participants, their data, or biological material

Policy information about studies with human participants or human data. See also policy information about sex, gender (identity/presentation), and sexual orientation and race, ethnicity and racism.

Reporting on sex and gender	No sex and gender-based analysis were carried out in this study.
Reporting on race, ethnicity, or other socially relevant groupings	No race, ethnicity, or other socially relevant variables were collected.
Population characteristics	A total of 61 HS images were acquired from 34 adult patients with brain tumours. Ages ranged from 30 to 73 years, with a median age of 51.5 years. Among these patients, there were 21 males and 13 females. Of these 34 patients, 28 (82.4%) had a primary tumour. The most frequency primary grade was the G4 (44.1%, n=15), followed by G1 and G2 (14.7%, n=5 each one), while the 8.8% (n=3) of the tumours were G3. The remaining 6 (17.6%) tumours were secondary: 3 from breast carcinoma, 2 from lung (one adenocarcinoma and one carcinoma), and 1 from kidney (renal carcinoma). Most of tumours were located in the right temporal lobe (23.5%, n=8), followed by the left frontal and right parietal lobes (20.6%, n=7 each).
Recruitment	All patients over 18 years of age, with primary or secondary brain tumours, undergoing brain surgery at the University Hospital of Gran Canaria Doctor Negrín (Spain) who were capable of giving informed consent for this study protocol before the surgery. Patients were enrolled in three different data acquisition campaigns carried out between March 2015 to June 2016 (First Campaign), October 2016 to April 2017 (Second Campaign) and July 2019 to October 2019 (Third Campaign).
Ethics oversight	Written informed consent was obtained from all the participant subjects. The study protocol and consent procedures were approved by the Research Ethics Committee of the University Hospital Doctor Negrin (Ref 130069 for the first and second campaigns, and Ref 2019-001-1 for the third campaign). All the research methodologies were performed in accordance with relevant guidelines/regulations.

Note that full information on the approval of the study protocol must also be provided in the manuscript.

Field-specific reporting

Please select the one below that is the best fit for your research. If you are not sure, read the appropriate sections before making your selection.

☒ Life sciences ☐ Behavioural & social sciences ☐ Ecological, evolutionary & environmental sciences

For a reference copy of the document with all sections, see nature.com/documents/nr-reporting-summary-flat.pdf

Life sciences study design

All studies must disclose on these points even when the disclosure is negative.

Sample size	No sample-size calculation was performed since we collected as much data as possible for the training, validation and testing of the processing algorithms developed in this work at technical validation level.
Data exclusions	24 HS images were excluded due to inadequate capturing conditions and were not employed in the data analyses.
Replication	The experiments performed in this work were reproducible when using the same data partitions. All attemps at replication were succesful.
Randomization	To correctly evaluate the classification performance of the proposed approach, a three-way data partition was carried out at patient level, dividing the HS database into training (60%), validation (20%), and test (20%) sets. Additionally, five different folds were created to achieve more robust results due to the limited number of patients. This data partition was performed randomly using the patients' identifiers as instances, where each patient could have more than one HS image. Labelled data were employed to train the classification models (training set), to optimize their hyperparameters (validation set), and to quantitatively evaluate the results using unseen HS data (test set). The hyperparameter optimization of each algorithm was performed in each fold independently, evaluating the results with their respective validation sets and using the macro F1-Score metric and performing a coarse search. The optimal hyperparameters were selected using the best macro F1-Score result of each fold without considering the background class.
Blinding	The blinded process was not relevant for this study, since randomized HS data at patient level was provided to the processing algorithms.

Reporting for specific materials, systems and methods

We require information from authors about some types of materials, experimental systems and methods used in many studies. Here, indicate whether each material, system or method listed is relevant to your study. If you are not sure if a list item applies to your research, read the appropriate section before selecting a response.

Materials & experimental systems

n/a	Involved in the study
☒	☐ Antibodies
☒	☐ Eukaryotic cell lines
☒	☐ Palaeontology and archaeology
☒	☐ Animals and other organisms
☐	☒ Clinical data
☒	☐ Dual use research of concern
☒	☐ Plants

Methods

n/a	Involved in the study
☒	☐ ChIP-seq
☒	☐ Flow cytometry
☒	☐ MRI-based neuroimaging

Clinical data

Policy information about clinical studies

All manuscripts should comply with the ICMJE guidelines for publication of clinical research and a completed CONSORT checklist must be included with all submissions.

Clinical trial registration	The study protocol and consent procedures were approved by the Research Ethics Committee of the University Hospital Doctor Negrin (Ref 130069 for the first and second campaigns, and Ref 2019-001-1 for the third campaign).
Study protocol	Study protocol is not publicly available.
Data collection	Data was collected at the University Hospital of Gran Canaria Doctor Negrín (Spain) in three different data acquisition campaigns carried out between March 2015 to June 2016 (First Campaign), October 2016 to April 2017 (Second Campaign) and July 2019 to October 2019 (Third Campaign).
Outcomes	Data collection was only carried out to train, validate and test the processing algorithms developed at technical validation level. Any outcomes were measured in the subjects involved in the study for collecting HS data.
